# Leveraging Systematic Reviews to Explore Disease Burden and Costs of Per- and Polyfluoroalkyl Substance Exposures in the United States

**DOI:** 10.1007/s12403-022-00496-y

**Published:** 2022-07-26

**Authors:** Vladislav Obsekov, Linda G. Kahn, Leonardo Trasande

**Affiliations:** 1 Department of Pediatrics, NYU Grossman School of Medicine, New York, NY USA; 2 Department of Population Health, NYU Grossman School of Medicine, New York, NY USA; 3 Department of Environmental Health, NYU Grossman School of Medicine, New York, NY USA; 4 NYU Wagner School of Public Service, New York, NY USA; 5 NYU School of Global Public Health, New York, NY USA

**Keywords:** PFAS, Perfluoroalkyl substances, Polyfluoroalkyl substances, Environmental chemicals, Disease burden, Economic costs, Obesity, Diabetes, Metabolism, Cancer, Reproductive health, Fertility, Respiratory infection, Child health

## Abstract

Accelerating evidence confirms the contribution of per- and polyfluoroalkyl substances (PFAS) to disease burden and disability across the lifespan. Given that policy makers raise the high cost of remediation and of substituting PFAS with safer alternatives in consumer products as barriers to confronting adverse health outcomes associated with PFAS exposure, it is important to document the costs of inaction even in the presence of uncertainty. We therefore quantified disease burdens and related economic costs due to legacy PFAS exposures in the US in 2018. We leveraged systematic reviews and used meta-analytic inputs whenever possible, identified previously published exposure–response relationships, and calculated PFOA- and PFOS-attributable increases in 13 conditions. These increments were then applied to census data to determine total annual PFOA- and PFOS-attributable cases of disease, from which we calculated economic costs due to medical care and lost productivity using previously published cost-of-illness data. We identified PFAS-attributable disease costs in the US of $5.52 billion across five primary disease endpoints shown to be associated with PFAS exposure in meta-analyses. This estimate represented the lower bound, with sensitivity analyses revealing as much as $62.6 billion in overall costs. While further work is needed to assess probability of causation and establish with greater certainty effects of the broader category of PFAS, the results confirm further that public health and policy interventions are still necessary to reduce exposure to PFOA and PFOS and their endocrine-disrupting effects. This study demonstrates the large potential economic implications of regulatory inaction.

**Supplementary Information:**

The online version contains supplementary material available at 10.1007/s12403-022-00496-y.

## Introduction

Per- and polyfluoroalkyl substances (PFAS) are a group of over 4700 human-made fluorine-rich molecules (Birnbaum 2018). Long-chain PFAS, with a minimum of six carbons in their “backbone,” were first developed in the 1940s. The polarity of their structure enhanced their utility in the production of water- and oil-resistant clothing, electronics, nonstick cookware, carpets, and food packaging materials for many years (Arbuckle et al. 2013; Holzer et al. 2008). These chemicals are widely detected in the blood of human populations worldwide (Bach et al. 2016a; Calafat et al. 2007), in part due to the biological persistence of many long-chain PFAS, such as perfluorooctanoic acid (PFOA) and perfluorooctane sulfonic acid (PFOS), which have half-lives in humans of at least 2 years (Bartell et al. 2010; Olsen et al. 2007; Xu et al. 2020). Although PFOA and PFOS have been added to the Stockholm Convention and PFOA use has been banned in the EU, they are still being released into the environment and are still being produced in other countries. Furthermore, both chemicals persist in the environment due to their chemical stability, resulting in ongoing human exposure (Grandjean and Clapp 2015).

Among the first to document PFAS-related effects on human health were the C8 Science Panel exposure and health studies conducted between 2005 and 2013 in mid-Ohio Valley communities where PFOA had heavily contaminated the water supply since the 1950s. These studies identified probable links with diagnosed high cholesterol, ulcerative colitis, thyroid disease, testicular cancer, kidney cancer, and pregnancy-induced hypertension (C8 Science Panel). An updated report from C8 Science Panel members and colleagues suggests that while the epidemiologic evidence for some of the associations they identified remains limited, possibly due to lower exposure levels in the general population, their findings for high cholesterol, ulcerative colitis, and kidney and testicular cancer had been reinforced by subsequent studies and impaired immune function had emerged as an additional outcome (Steenland et al. 2020). A recent scoping review of studies exclusively conducted among general population samples concluded that the weight of evidence supported associations of low-level PFAS exposure with low birth weight (LBW, < 2500 g), childhood obesity, adult obesity, adult-onset type 2 diabetes (T2D), gestational diabetes (GDM), endometriosis, polycystic ovarian syndrome (PCOS), couple infertility, and breast cancer (Kahn et al. 2020). Systematic reviews add further support for routine PFAS exposure and LBW (Bach et al. 2015; Johnson et al. 2014; Koustas et al. 2014; Lam et al. 2014; Steenland et al. 2018); childhood obesity (Liu et al. 2018b), dyslipidemia (Rappazzo et al. 2017), renal dysfunction (Rappazzo et al. 2017), respiratory infection (Rappazzo et al. 2017), and reduced immune response to vaccines (Rappazzo et al. 2017); age at menarche (Rappazzo et al. 2017); and adult thyroid dysfunction (Kim et al. 2018) and kidney (Bartell and Vieira 2021), testicular (Bartell and Vieira 2021), and breast (Wan et al. 2021) cancers. While a 2016 systematic review cast doubt on evidence for infertility due to PFAS because most of the studies that found associations were not restricted to nulliparous women (Bach et al. 2016b), the authors acknowledged that four of eight studies identified increased time to pregnancy (TTP) with PFOA or PFOS exposure. A more recent scoping review was less dismissive of the evidence and pointed out that studies conducted among parous women may still be valid if models adjust for interpregnancy interval and (in retrospective studies) gestational age at blood collection (Kahn et al. 2021). A difference-in-difference analysis of a natural experiment in which fertility and birth outcomes were compared between communities without PFAS exposure and highly exposed communities where PFOA- and PFOS-contaminated water supplies were remediated found that preterm birth and LBW rates, which had been higher in the contaminated communities, decreased following remediation and the fertility rate, which had been lower, increased (Waterfield et al. 2020).

In the United States (US), rising concerns about the health effects of PFAS have prompted calls to state and federal governments to limit ongoing PFAS use and remediate contaminated water supplies. The US Environmental Protection Agency’s third Unregulated Containment Monitoring Rule report released in January 2017 found that 4% of water systems reported at least one PFAS compound detectable above the minimum reporting level, which ranged from 10 to 90 parts per trillion (ppt) for various PFAS (Crone et al. 2019). A more recent study estimates that 18–80 million people in the US receive tap water with at least 10 ppt of PFOA and PFOS combined and more than 200 million Americans have tap water contaminated with PFOA and PFOS concentrations of 1 ppt or higher (Andrews and Naidenko 2020). Although there is currently no national regulatory limit for PFOA and PFOS exposure and the US Environmental Protection Agency continues to use a lifetime health advisory level of 70 ppt for the sum of PFOA and PFOS (Environmental Protection Agency 2016), some states have banned PFAS in food packaging and lowered regulatory limits for PFAS in drinking water by two orders of magnitude to 1 ppt or lower as suggested by studies of PFAS and antibody titers in children (Grandjean and Clapp 2015; Hoylman 2020).

In considering regulatory action, the European Food Safety Authority and the US Agency for Toxic Substances and Disease Registry have suggested that the evidence is not sufficient to confirm causality and therefore to proceed with steps to reduce exposure (Rogers et al. 2021; Schrenk et al. 2020). As Bradford Hill declared in his landmark lecture on causal inference (Hill 1965), uncertainty “does not confer upon us a freedom to ignore the knowledge we already have or to postpone the action that it appears to demand at a given time.” Given that policy makers raise the high cost of remediation and of substituting PFAS with safer alternatives in consumer products as barriers to confronting adverse health outcomes associated with PFAS exposure, it is important to document the costs of inaction even in the presence of uncertainty.

Recent studies suggest that the disease-related burden due to PFAS can be substantial. In 2003–2004, PFOA exposure accounted for up to 4% of LBW in the US, with $13.7 billion in associated costs (Malits et al. 2018). The Nordic Council of Ministers estimated €52–84 billion in disease-related costs in 2019 associated with PFAS within the European Economic Area, driven substantially by occupational PFAS exposures and effects on populations living near contaminated sites (Goldenman et al. 2019). Yet, these analyses did not consider the broader scope of health effects of routine, low-level environmental PFAS exposure on human health. The aim for this analysis was therefore to quantify disease burden and associated costs of PFOA and PFOS exposure among the entire US population based on health outcomes with a substantial weight of evidence in support of associations with PFAS exposure.

## Methods

### ***Overall approach (Fig. ******1******)***

**Fig. 1:**
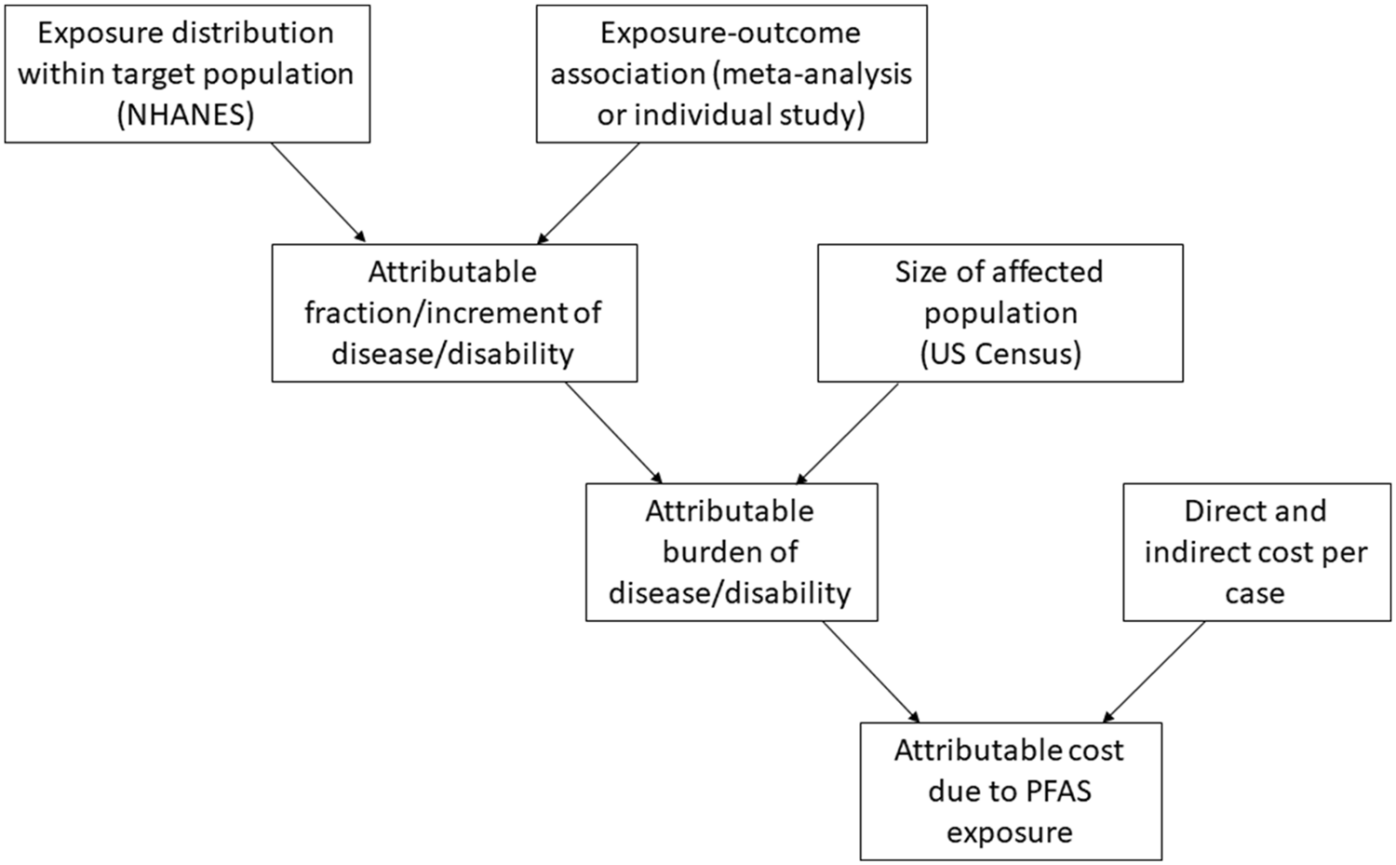
Schematic of method for calculating cost of disease/disability attributable to PFAS exposure

To identify diseases and dysfunctions and their associated economic costs to be considered for possible attribution to PFAS exposure, we leveraged the PFAS-Tox Database (https://pfastoxdatabase.org/), which was built using systematic review methods, to extract relevant studies and data (Pelch et al. 2019). Given the rapidly evolving nature of the PFAS literature, we supplemented the primary source with a PubMed search using the terms “PFAS” and “systematic review” or “meta-analysis.” In an additional effort to be complete, we also mined three recent scoping reviews to ensure the most comprehensive inclusion of potential disease burden and costs in sensitivity analyses (Kahn et al. 2020; Steenland et al. 2020; Steenland and Winquist 2021).

In main estimates of PFAS-attributable disease burden and cost, we only considered disease outcomes for which statistically significant associations had been derived from published meta-analyses of epidemiologic studies. These included (1) LBW due to prenatal exposure (Steenland et al. 2018); (2) childhood obesity due to prenatal exposure (Liu et al. 2018b); (3) kidney cancer due to lifetime exposure (Bartell and Vieira 2021); (4) testicular cancer due to lifetime exposure (Bartell and Vieira 2021); and (5) hypothyroidism in females due to lifetime exposure (Kim et al. 2018). For all of these outcomes except hypothyroidism, we used the meta-analytic estimates of exposure–response relationship [ERR, e.g., odd ratios (ORs) or risk ratios (RRs)] as the bases for disease burden and cost estimations. For hypothyroidism, a clear negative association was demonstrated between PFOA exposure and total T4 and T3 levels in a meta-analysis of seven papers by Kim et al., but because these are not clinical endpoints, we used an OR from a representative paper from the meta-analysis that identified a negative association with subclinical hypothyroidism (Wen et al. 2013).

In a sensitivity analysis of PFAS-attributable disease burden and cost, we expanded the scope of relevant outcomes to consider health conditions for which relations with PFAS had been identified in systematic and scoping reviews but had not been meta-analyzed. These included (6) adult obesity due to exposure over the lifespan (Kahn et al. 2020); (7) T2D in females due to exposure over the lifespan (Kahn et al. 2020); (8) GDM due to exposure measured in pregnancy (Kahn et al. 2020); (9) endometriosis due to exposure over the lifespan (Kahn et al. 2020); (10) PCOS due to exposure over the lifespan (Kahn et al. 2020); (11) couple infertility due to lifetime exposure in females (Kahn et al. 2020); (12) female breast cancer due to lifetime exposure (Wan et al. 2021); and (13) pneumonia in children due to prenatal exposure (Rappazzo et al. 2017). We did not include pediatric dyslipidemia or reduced age at menarche (Rappazzo et al. 2017), as these indicators are associated with outcomes already included in our analysis (e.g., childhood obesity and breast cancer, respectively); similarly, we did not include adult high cholesterol (Steenland et al. 2020), as it is associated with adult obesity, which is already included in our analysis. We also did not include reduced response to childhood vaccination (Grandjean et al. 2012), as reduced titers generally only require revaccination and clinical episodes of tetanus [~ 30 cases per year (CDC)] and diphtheria [2 cases between 2004 and 2017 (CDC)] are extremely rare in the US (CDC 2022a, b). Recognizing that some studies for each of the included outcomes might have reported null findings, the lower bound of economic cost added for this group of outcomes is zero. We based the upper bound of the sensitivity analysis on ERRs drawn from recent well-designed studies that reported statistically significant results from populations most similar to the current US population and extracted appropriate ERRs for our exposures and outcomes of interest (Tables 1, 2). To extrapolate most accurately effects in 2018 [the most recent year for which PFAS exposure data are available from the US National Health and Nutrition Examination Survey (NHANES)], we considered only studies published within the past 10 years and excluded those that did not control for confounding variables in the analysis, did not have PFAS exposure levels similar to our population as defined by the 2017–2018 NHANES dataset, and did not provide an RR, OR, or beta coefficient with either a 95% confidence interval or *p*-value. When multiple studies met these criteria, we modeled each separately and added the highest estimate to our cost estimate total in order to establish the upper bound of our sensitivity analysis.

**Table 1:** Study selection

Exposure	Author (Year)	N	Study type	Date of recruitment	Location	Population	Exposure	Outcome	Covariates	Results
Low birth weight										
PFOA~	Steenland (2018)	24 studies	Meta-analysis	Varied by study	Varied by study	Varied by study	PFOA in maternal or cord blood	Birth weight	Varied by study	10.5 g (4.4, 16.7) decrease in birth weight per ng/mL PFOA increase in maternal or cord blood
PFOS*	Meng (2018)	3535 mother–infant pairs	Cross-sectional	1992–2002	Denmark	Mother–infant pairs	PFOS in maternal plasma	Birth weight	Infant sex, infant birth year, gestational week of blood draw, maternal age, parity, socio-occupational status, pre-pregnancy BMI, smoking, and alcohol use during pregnancy	45.2 g (13.6, 76.8) decrease in birth weight per doubling of ng/mL PFOS increase in maternal plasma
Childhood obesity at age 10										
PFOA~	Liu (2018a)	Nine studies with prenatal exposure	Meta-analysis	Varied by study	Varied by study	Multiple cohorts of children	PFOA in early childhood	BMI	Varied by study	0.09 (0.02, 0.17) increase in BMI *z*-score per ng/mL increase in prenatal PFOA
PFOS*	Lauritzen (2018)	412 females	Prospective cohort	1986–1988	Norway and Sweden	Pregnant women	PFOS in maternal serum	BMI	Maternal age, education, smoking at conception, pre-pregnancy BMI, weight gain at 17 weeks, interpregnancy interval, previous breastfeeding duration, and country of residence	0.18 (0.01, 0.35) increase in BMI *z*-score per ln-unit ng/mL increase in prenatal PFOA
Kidney cancer										
PFOA~	Bartell (2021)	Four studies	Meta-analysis	Varied by study	Varied by study	Varied by study	PFOA exposure	Kidney cancer incidence	Varied by study	Increase in cancer risk per 10 ng/mL increase in serum PFOA = 16% (3%, 30%)
Testicular cancer										
PFOA~	Bartell (2021)	Two studies	Meta-analysis	Varied by study	Varied by study	Varied by study	PFOA exposure	Testicular cancer incidence	Varied by study	Increase in cancer risk per 10 ng/mL increase in serum PFOA = 3% (2%, 4%)
Hypothyroidism										
PFOA~	Kim (2018)	Seven studies after excluding outliers	Cross-sectional	2007–2009	USA	Adults > 20 years old	PFOA in serum	Subclinical hypothyroidism in women	Age, race, drinking, smoking, and natural log-urinary iodine	7.42 (1.14–48.12) OR of subclinical hypothyroidism risk per ln-unit ng/mL increase in serum PFOA
PFOS*	Wen (2013)	1181 individuals	Cross-sectional	2007–2009	USA	Adults > 20 years old	PFOS in serum	Subclinical hypothyroidism in women	Age, race, drinking, smoking, and natural log-urinary iodine	3.03 (1.14–8.07) OR of subclinical hypothyroidism risk per ln-unit ng/mL increase in serum PFOA
Adult obesity										
PFOS*	Liu (2018a)	520 individuals	Randomized clinical trial	2003–2007	Boston, MA and Baton Rouge, LA	Over-weight and obese 30–70-year olds	PFOS in serum	Body weight	Age, sex, race, baseline BMI, education, smoking status, alcohol consumption, physical activity, and dietary intervention group	Higher baseline levels of PFOS associated with greater weight regain (1.5 ± 0.6–3.2 ± 0.6 kg)
Adult-onset type 2 diabetes										
PFOA*	Sun (2018)	1586 females	Prospective nested case–control study	1995–2000	USA	Female nurses 32–52 years old	PFAS in plasma	T2D	Age, month, and fasting status at sample collection and menopausal status and hormone replacement therapy	1.54 (1.04–2.28) OR of T2D in highest tertile of exposure compared to lowest
Gestational diabetes										
PFOA*	Zhang (2015)	258 females	Prospective cohort	2005–2009	Michigan and Texas	Women discontinuing contraception to become pregnant	PFOA in serum	GDM	Age, BMI, and parity conditional on gravidity	1.61 (1.05–2.49) OR of GDM per SD increment of PFOA exposure
Endometriosis										
PFOA*	Buck Louis (2012)	626 females	Prospective nested case–control study	2007–2009	Salt Lake City, UT and San Francisco, CA	Women 18–44 years old	PFOA in serum	Endometriosis	Age, BMI, and parity	1.89 (1.17–3.06) OR of endometriosis per log unit exposure of PFOA
Polycystic ovarian syndrome										
PFOA*	Vagi (2014)	102 females	Case–control	2007–2008	Los Angeles, CA	Women 18–45 years old	PFOA in serum	PCOS	Age, BMI, and race	6.93 (1.79–29.92) OR of PCOS in highest tertile compared to lowest
PFOS*	Vagi (2014)	102 females	Case–control	2007–2008	Los Angeles, CA	Women 18–45 years old	PFOS in serum	PCOS	Age, BMI, and race	5.79 (1.58–24.12) OR of PCOS in highest tertile compared to lowest
Couple infertility										
PFOA*	Bach (2015)	1601 females	Case–control	1996–2002	Denmark	Pregnant women	PFOA in serum	Time to pregnancy	Age, socio-economic status, BMI, and parity	0.67 (0.51–0.88) fecundability ratio per log unit PFOA exposure
PFOS*	Bach (2015)	1601 females	Case–control	1996–2002	Denmark	Pregnant women	PFOS in serum	Time to pregnancy	Age, socio-economic status, BMI, and parity	0.62 (0.47–0.83) fecundability ratio per log unit PFOS exposure
Breast cancer										
PFOA*	Wielsoe (2017)	161 females	Case–control	2000–2003, 2011–2014	Greenland	Inuit women	PFOA in serum	Breast cancer	Age, BMI, cotinine levels, parity, and breastfeeding	1.26 (1.01–1.58) OR of breast cancer with PFOA exposure
PFOS*	Wielsoe (2017)	161 females	Case–control	2000–2003, 2011–2014	Greenland	Inuit women	PFOS in serum	Breast cancer	Age, BMI, cotinine levels, parity, and breastfeeding	1.02 (1.01–1.03) OR of breast cancer with PFOS exposure
Pneumonia										
PFOA*	Impinen (2019)	1270 females	Cohort	1999–2008	Norway	Children	PFOA in maternal serum	Number of infections by age 3	Maternal age, maternal BMI, maternal education, parity, and smoking during pregnancy	1.27 (1.12–1.43) RR of bronchitis/pneumonia
PFOS*	Impinen (2019)	1270 females	Cohort	1999–2008	Norway	Children	PFOS in maternal serum	Number of infections by age 3	Maternal age, maternal BMI, maternal education, parity, and smoking during pregnancy	1.20 (1.07–1.34) RR of bronchitis/pneumonia

*Sensitivity analysis

^~^From meta-analyses

**Table 2:** Exposure–response relationships

Outcome	Exposed population	Exposure	Exposure modeling (ng/mL)	ERR	Source of ERR
Low birth weight~					
	Females 18–49	PFOA	Continuous	*β* = − 10.5 g/PFOA (ng/mL)	Steenland (2018)
		PFOS	Continuous	*β* = − 45.2 g/doubling of PFOS (ng/mL)	Meng (2018)
Childhood obesity at age 10~					
	Females 18–49	PFOA	Continuous	*β* = 0.09 per PFOA (ng/mL)	Liu (2018a)
		PFOS	Continuous	*β* = 0.18 per ln((PFOS) (ng/mL))	Lauritzen (2018)
Kidney cancer					
	Adults 18+	PFOA	Continuous	OR = 1.16 per 10 ng/mL PFOA	Bartell (2021)
Testicular cancer					
	Males 18+	PFOA	Continuous	OR = 1.03 per 10 ng/mL PFOA	Bartel (2021)
Hypothyroidism					
	Females 18–49	PFOA	Continuous	OR = 7.42 per ln(PFOA (ng/mL))	Wen (2013)
		PFOS	Continuous	OR = 3.03 per ln(PFOA (ng/mL))	
Adult obesity					
	Adults 18+	PFOS	Tertile 1: < 19.2	*β* = 1.5 kg gained	Liu (2018a)
			Tertile 2: 19.2–32.1	*β* = 3.5 kg gained	
			Tertile 3: > 32.1	*β* = 3.2 kg gained	
Adult-onset type 2 diabetes					
	Females 18–49	PFOA	Tertile 1: < 3.76	OR = 1	Sun (2018)
			Tertile 2: 3.76–5.48	OR = 1.27	
			Tertile 3: > 5.48	OR = 1.54	
Gestational diabetes					
	Females 18–49	PFOA	Continuous	When ln(1 + PFOA (ng/mL) increases by 1 SD, OR increases by 1.61	Zhang (2015)
Endometriosis					
	Females 18–49	PFOA	Continuous	OR = 1.89 per log((PFOA (ng/mL))	Buck Louis (2012)
Polycystic ovarian syndrome					
	Females 15–45	PFOA	Tertile 1: < 2.6	OR = 1	Vagi (2014)
			Tertile 2: 2.6–4.1	OR = 1.65	
			Tertile 3: > 4.1	OR = 6.93	
		PFOS	Tertile 1: < 6.2	OR = 1	
			Tertile 2: 6.2–8.6	OR = 3.43	
			Tertile 3: > 8.6	OR = 5.79	
Couple infertility					
	Females 18–49	PFOA	Continuous	OR = 0.67 per log((PFOA (ng/mL))	Bach (2015)
		PFOS	Continuous	OR = 0.62 per log((PFOS (ng/mL))	
Breast cancer					
	Females 18–49	PFOA	Continuous	OR = 1.26 per PFOA (ng/mL)	Wielsoe (2017)
		PFOS	Continuous	OR = 1.02 per PFOS (ng/mL)	
Pneumonia~					
	Females 18–49	PFOA	Continuous	RR = 1.27 per PFOA (ng/mL)	Impinen (2019)
		PFOS	Continuous	RR = 1.20 per PFOS (ng/mL)	

~ Serum concentrations in females of childbearing age was used as a proxy for prenatal exposure

### Assessing Risk of Bias

A substantial literature has described and compared methods to evaluate systematic reviews (Whiting et al. 2016) and epidemiologic studies (Eick et al. 2020) for risk of bias. We used the tool developed by the National Toxicology Program’s Office of Health Assessment and Translation (OHAT) (Office of Health Assessment and Translation (OHAT) 2022) to evaluate epidemiologic studies and ROBIS, the first rigorously developed tool designed specifically to assess the risk of bias in systematic reviews (Whiting et al. 2016). Two authors (LT, LK) independently evaluated each of the studies.

OHAT includes seven questions that yield graded probability assessments for risk of bias within observational studies (definitely low, probably low, probably high, definitely high). In the cases where there was a potential risk of bias, we added narrative comments to explain reasons for our concerns. ROBIS evaluates risk of bias in systematic reviews across four domains: study eligibility criteria; identification and selection of studies; data collection and study appraisal; and synthesis and findings. Within each domain, answers to multiple questions are used to assemble a domain-wide assessment of risk of bias (low, high, unclear). For each systematic review, two authors (LT and LK) assessed overall risk of bias in each of the four domains, identified specific concerns, and then assessed whether conclusions were supported by the evidence based on three criteria: whether the interpretation of findings addressed identified concerns in all the domains; whether the relevance of identified studies to the research question was appropriately considered; and whether authors overemphasized statistical significance. These questions were answered as yes, probably yes, probably no, no, or no information. This informed final assessments of each systematic review as low, high, or unclear.

### Estimating PFAS-Attributable Disease Burden and Cost

To estimate the attributable cost of PFAS-mediated disease, we applied the model first used by the Institute of Medicine (1981) described by the equations below:

1 $$ {\text{Attributable disease burden}} = {{\text{Increment in disease}} \mathord{\left/ {\vphantom {{\text{Increment in disease}} {{\text{disability}}}}} \right. \kern-\nulldelimiterspace} {{\text{disability}}}} \times {\text{Attributable fraction}}\left( {{\text{AF}}} \right) \times {\text{Population size}} $$

2 $$ {\text{Attributable cost}} = {\text{Attributable disease burden}} \times {\text{Cost per increment}}. $$

The AF of a risk factor can be defined as the proportional decrease in the number of cases of ill health or deaths as a result of reducing the risk factor to a reference level and can be estimated using the following equation:

3 $$ {\text{AF}} = {\text{Prevalence}}_{{{\text{exposure}}}} *{{\left( {{\text{relative risk}}\left( {{\text{RR}}} \right) - {1}} \right)} \mathord{\left/ {\vphantom {{\left( {{\text{relative risk}}\left( {{\text{RR}}} \right) - {1}} \right)} {\left[ {{1} + \left( {{\text{prevalence}}_{{{\text{exposure}}}} *\left( {{\text{RR}} - {1}} \right)} \right)} \right]}}} \right. \kern-\nulldelimiterspace} {\left[ {{1} + \left( {{\text{prevalence}}_{{{\text{exposure}}}} *\left( {{\text{RR}} - {1}} \right)} \right)} \right]}}, $$

where RR represents the risk of morbidity associated with the specific exposure relative to the reference level (Levin 1953).

The first step in calculating attributable disease burden was to quantify exposure. We focused our analysis on PFOA and PFOS, as they are the most widely studied members of the PFAS class and evidence of their health effects is strongest. Because these two chemicals co-occur, as a conservative measure, we calculated disease burden based on PFOA and PFOS separately as proxies for long-chain PFAS exposure. Our source for distributions of exposure was the 2017–2018 cycle of NHANES, as this contains the most recent nationally representative data. For each analysis, we focused on the relevant subsection of the population (e.g., women of childbearing age when considering PCOS). For childhood obesity we used data from the 2007–2008 cycle to quantify in utero exposure among children who were age 10 in 2017–2018. NHANES measured serum concentrations of PFOA and PFOS with online solid-phase extraction coupled with high-performance liquid chromatography–tandem mass spectrometry; an extensive methodology is provided in the NHANES Laboratory Procedures Manual (CDC 2016) Main estimates used PFOA levels, while sensitivity analyses considered PFOS levels, as well.

We stratified the US population into percentile groupings of serum PFAS concentration (< 10th, 10th–24th, 25th–49th, 50th–74th, 75th–89th, 90th–99th, and > 99th). As a conservative measure, we assumed exposures within each percentile grouping to be at the lowest end of the range (e.g., corresponding to the 10th, 25th, 50th, 75th, 90th, and 99th percentile) and assumed no exposure for the lowest 10% of the population, our reference group (Table 3).

**Table 3:** NHANES exposure data

Outcome	Exposed population	NHANES years of exposure	Exposure chemical	Concentrations assigned to each percentile of exposure						
				0–9	10–24	25–49	50–74	75–89	90–99	> 99
Low birth weight*, adult diabetes, gestational diabetes, endometriosis, couple infertility, breast cancer, pneumonia*, and hypothyroidism										
	Females 18–49	2017–2018	PFOA (ng/mL)	0.00	0.47	0.67	0.97	1.47	2.37	5.17
			PFOS (ng/mL)	0.00	1.10	1.70	2.60	3.90	5.70	11.9
Childhood obesity at age 10*										
	Females 18–49	2007–2008	PFOA (ng/mL)	0.00	2.30	2.70	3.50	4.60	5.90	7.10
			PFOS (ng/mL)	0.00	4.70	7.20	9.90	17.70	24.30	32.20
Adult obesity and kidney cancer										
	Adults 18+	2017–2018	PFOA (ng/mL)	0.00	0.67	0.97	1.47	2.17	3.07	8.30
			PFOS (ng/mL)	0.00	1.60	2.70	4.70	7.80	12.0	26.2
Polycystic ovarian syndrome										
	Females 15–45	2017–2018	PFOA (ng/mL)	0.00	0.47	0.67	0.87	1.37	2.07	5.17
			PFOS (ng/mL)	0.00	1.10	1.70	2.50	3.60	5.30	10.7
Testicular cancer										
	Males 18+	2017–2018	PFOA (ng/mL)	0.00	0.87	1.17	1.67	2.27	3.27	8.30
			PFOS (ng/mL)	0.00	2.60	3.60	5.80	9.20	13.0	26.2

*Serum concentrations in females of childbearing age were used as a proxy for prenatal exposure

Once we established the exposure level across each percentile group, we calculated increments in disease or disability over the baseline population rate due to exposure. 2018 US Census estimates (USC Bureau 2020) were used to convert the baseline prevalence or incidence values to the appropriate population size (subsequent sections identify the sources of prevalence/incidence data for each outcome). We then applied the previously selected ERRs to quantify attributable burdens of disease within each group. If an ERR was based on continuous exposure in the literature, main analyses employed a reference level of 0.1 ng/mL below which no effects were assumed to be observed. If an ERR was based on tertiles or quartiles of exposure in the literature, we used the lowest quantile as the reference level (Table 3). ORs were converted to RRs to avoid overestimation following published practice (Knol et al. 2012), and Levin’s formula was used to tabulate AFs based on RRs (Levin 1953).

Once we estimated the increase in cases attributable to PFOA/PFOS exposure for the 13 outcome measures, we calculated associated economic costs using available data on cost per case, derived from previously published estimates of direct and/or indirect healthcare and societal costs, and the size of the population at risk (Supplementary Tables 1–13). All cost estimates were adjusted to reflect the annual average for 2018 in US dollars using the All Items Consumer Price Index (US Bureau of Labor Statistics 2020).

### Sensitivity Analyses

As our main result, we reported the PFOA disease burden and cost estimates for the five disease outcomes with meta-analytic associations and then summed them. We then generated alternative estimates through multiway sensitivity analyses to provide the most accurate range of possible costs (Table 4). First, we calculated disease burden and cost estimates using ERRs for PFOS and serum levels from NHANES for the same group of outcomes. We then calculated disease burden and cost estimates for PFOA using an expanded group of outcomes that included both those conditions for which there were meta-analytic results and those for which there were results from systematic or scoping reviews. We also examined the influence of a higher reference level (1.0 ng/mL) on disease burden and costs for which ERRs were based on continuous exposure. Finally, we repeated this analysis for the expanded group of conditions, substituting PFOS for PFOA. The boundaries of the sensitivity analysis were identified using the lowest and highest values for each of the adverse endpoints studied, which were aggregated to create a range for probable disease costs due to PFAS.

**Table 4:** Total disease burden and costs in 2017–2018

	Primary analysis	Sensitivity analysis	
	Main estimate from meta-analyses	Low estimate	High estimate
Low birth weight			
Attributable incident cases per year	10,053	–	96,847
Attributable fraction	3.17%	–	30.7%
Total cost per annual incident case	$1,420,000,000	–	$13,700,000,000
Direct cost of hospitalization	$305,000,000	–	$2,940,000,000
Indirect cost due to lost IQ points	$1,110,000,000	–	$10,700,000,000
Childhood obesity at age 10			
Attributable incident cases per year	127,362	–	462,119
Attributable fraction	3.78%	–	13.70%
Incremental lifetime medical cost of an obese child relative to normal weight child due to annual incident cases	$2,650,000,000	–	$9,600,000,000
Kidney cancer			
Attributable incident cases per year	142	–	–
Attributable fraction	0.34%	–	–
Total cost per annual incident case	$184,000,000	–	–
Direct medical cost during 1st year of diagnosis	$4,740,000	–	–
Indirect cost as DALY lost over 10 years	$180,000,000	–	–
Testicular cancer			
Attributable incident cases per year	5	–	–
Attributable fraction	0.076%	–	–
Total cost per annual incident case	$6,850,000	–	–
Direct medical cost of treatment	$139,000	–	–
Indirect cost as DALY lost over 10 years	$6,710,000	–	–
Hypothyroidism in females			
Attributable incident cases per year	14,572	–	59,939
Attributable fraction	5.0%	–	20.7%
Total cost per annual incident case	$1,260,000,000	–	$5,180,000,000
Direct cost of new cases of hypothyroidism annually	$42,100,000	–	$173,000,000
Indirect cost as DALY lost over 10 years	$1,220,000,000	–	$5,000,000,000
Adult obesity			
Attributable incident cases per year	–	4,294,379	–
Attributable fraction	–	2.98%	–
Total 15-year cost per annual incident case	–	$17,000,000,000	–
Direct medical cost for newly obese 35-year olds	–	$3,210,000,000	–
Indirect cost of QALY lost over 15 years	–	$13,800,000,000	–
Adult type II diabetes in females			
Attributable incident cases per year	–	1728	–
Attributable fraction	–	0.37%	–
Lifetime cost of treating type II diabetes and associated complications due to annual incident cases	–	$140,000,000	–
Gestational diabetes			
Attributable incident cases per year	–	6061	12,474
Attributable fraction	–	2.85%	5.87%
Total cost per annual incident case	–	$414,000,000	$852,000,000
Direct medical cost	–	$73,300,000	$150,000,000
Indirect cost of lost productivity from adverse birth effects	–	$341,000,000	$702,000,000
Endometriosis			
Attributable incident cases per year	–	696	18,062
Attributable fraction	–	0.43%	11.27%
Total 10-year cost per annual incident case	–	$397,000,000	$10,200,000,000
Direct medical cost over 10 years	–	$21,100,000	$547,000,000
Indirect cost as DALY lost over 10 years	–	$376,000,000	$9,760,000,000
Polycystic ovarian syndrome			
Attributable incident cases per year	–	7209	7505
Attributable fraction	–	5.92%	6.16%
Annual cost of initial PCOS evaluation and treatment of comorbidities due to annual incident cases	–	$10,500,000	$10,900,000
Couple infertility			
Attributable cases of ART SET utilized per year	–	593	26,160
Attributable fraction	–	0.25%	10.86%
Cost of attributable ART SET utilization per annual incident case	–	$37,600,000	$1,660,000,000
Breast cancer			
Attributable incident cases per year	–	421	3095
Attributable fraction	–	0.50%	3.65%
Total 10-year cost per annual incident case	–	$555,000,000	$4,080,000,000
Direct medical cost for 6 months following diagnosis per annual incident cases	–	$21,700,000	$159,000,000
Indirect cost as DALY lost over 10 years	–	$533,000,000	$3,920,000,000
Pneumonia			
Attributable incident cases per year in children 0–3 years old	–	447	6759
Attributable fraction	–	0.58%	8.81%
Total cost per incident case of pneumonia in 0–3-year olds	–	$1,490,000	$22,500,000
Direct medical cost of case across all healthcare settings	–	$1,320,000	$20,000,000
Indirect cost of parental absenteeism	–	$166,000	$2,510,000
Total cost	$5.52 billion	($5.52 billion–$62.6 billion)	

The following sections elaborate details of our methods specific to each disease outcome.

### Low Birth Weight

We updated a previously published approach to quantifying PFAS-attributable LBW (Malits et al. 2018) to include new literature and an estimate for PFOS, which we used in a sensitivity analysis. Briefly, we compared observed LBW in 2017–2018 to LBW in a counterfactual scenario in which PFOA/PFOS-attributable reductions in birth weight were eliminated, with the difference representing PFOA/PFOS-attributable LBW. For each 1.0 ng/mL of PFOA exposure above 0.1 ng/mL, a 10.5 g decrease [95% confidence interval (CI) − 16.7, − 4.4] in birth weight was applied in main analyses, based on the results of an updated meta-analysis (Steenland et al. 2018). Sensitivity analyses applied the lower 3.3 g decrement identified in a subset of studies with later pregnancy measures. For PFOS, we applied a 45.2 g decrease (95% CI − 76.8, − 13.6) in birth weight per doubling of early pregnancy maternal plasma concentrations from a study of 3535 mother–infant pairs in the Danish National Birth Cohort study (Meng et al. 2018). We used natality data from the National Vital Statistics System of the National Center for Health Statistics (CDC/NCHS 2014, 2018) to determine the actual mean birth weight, total number of births, and number of LBW births for 2017 and 2018 (the exposed scenario) and then increased mean birth weight in each PFOA/PFOS centile by the absolute value of the attributable decrement to calculate the number of LBW births in a scenario free of PFOA/PFOS effects. The PFOA/PFOS-attributable LBW disease burden was the difference in LBW births between the two, assuming a normal distribution of birth weight (Table S1). The average of results for 2017 and 2018 was calculated to represent PFAS-attributable LBW in 2018.

We calculated the total cost of LBW attributable to in utero PFAS exposure by adding the LBW-associated costs of hospitalization for medical concerns (direct cost) to the lost lifetime economic productivity, operationalized as loss of IQ points due to LBW (indirect cost). The direct cost of hospitalization was estimated at $30,364 per case in 2018 (Kowlessar et al. 2011). LBW has been associated with a 4.98 point loss in IQ (95% CI 3.20, 6.77) (Kormos et al. 2014). Applying a 3% discount rate for lifetime earnings, each IQ point loss was valued at $22,190 in 2018 (Gould 2009; Max et al. 2004). Both costs were multiplied by the number of additional LBW babies born over the 2 years attributable to PFOA and PFOS exposure to get the total cost.

### Childhood Obesity

We first quantified changes in body mass index (BMI) *Z*-score in subpopulations of children with increasing prenatal PFOA exposure by applying results of a meta-analysis of ten cohort studies, which identified a 0.09 increase in BMI *Z*-score for each ng/mL increase in PFOA (Liu et al. 2018b). For PFOS, we utilized a cohort study of 412 Norwegian and Swedish mother–infant pairs in which a 1 ng/mL increase in maternal serum levels was associated with 0.18 increase in BMI *Z*-score (Lauritzen et al. 2018). A 0.1 ng/mL reference level was applied in all analyses, below which no effects on BMI *Z*-score were included. The distribution of PFOA and PFOS in US women age 18–49 years in 2007–2008 was used as a proxy for the distribution in pregnant women during that time period. To estimate increases in childhood obesity in 10-year olds due to prenatal PFOA/PFOS exposure, we calculated increases in BMI *Z*-score and quantified incremental increases in *Z* > 1.64 (95th percentile).

Incremental increases in obesity were calculated from PFOA/PFOS-attributable increases in BMI *Z*-score using the NORMDIST function in Excel, assuming a mean = 0 and standard deviation (SD) = 1 without exposure. Increases in percent obese individuals were then multiplied by the number of 10-year olds in 2018 identified in US Census population estimates (Table S2) (USC Bureau 2020; Hales et al. 2017). We calculated the economic burden of PFOA/PFOS-attributable cases of childhood obesity based on an estimated lifetime medical cost of childhood obesity at age 10 of $20,780 in 2018 dollars (Finkelstein et al. 2014).

### Kidney Cancer

We utilized the ERR from Bartell et al. to identify the PFOA-attributable increased odds of kidney cancer based on a pooled increased risk of 16% per 10 ng/mL of PFOA exposure from a meta-analysis of four papers demonstrating the link between PFOA and kidney cancer (Bartell and Vieira 2021). We calculated ORs for PFOA in each percentile grouping based upon exposure levels in NHANES 2017–2018, applying a reference level of 0.1 ng/mL below which we assumed there was no increase in odds of kidney cancer. We then converted the ORs to RRs and adjusted for a kidney cancer prevalence of 12.89 per 10,000 adults in the US (Surveillance Research Program Surveillance, Epidemiology, and End Results Program). Afterward, we weighted the RRs by exposure percentile to calculate the AFs across all exposure percentiles using Levin’s equation (Levin 1953). The population incidence of kidney cancer, 16.9 per 100,000 adults/year, was obtained from the Surveillance, Epidemiology, and End Results Program and multiplied by the AF across the modeled range of population exposures and the US Census population estimates of the annual average number of adults over age 18 years in 2018 to quantify incident cases of kidney cancer attributable to PFOA exposure (Table S3) (Surveillance Research Program Surveillance, Epidemiology, and End Results Program).

Each case of newly diagnosed kidney cancer was associated with direct medical expenses of $33,485 in the first year alone in 2018 (Shih et al. 2019). We multiplied the cost by the PFOA-attributable cases for a total direct cost of first-year medical expenses for newly diagnosed kidney cancer in American adults. We then calculated the indirect 10-year cost of kidney cancer as lost disability-adjusted life years (DALY, 0.288 for each year, valued at $50,000/year) over 10 years, discounting 3% per year for future preference (Neumann et al. 2014). The total 10-year cost for a case of kidney cancer is the sum of first-year medical expenses and accrued indirect costs (DALY loss).

### Testicular Cancer

Similarly to kidney cancer, we utilized an ERR from Bartell et al. that demonstrated a 3% increase in risk of testicular cancer per 10 ng/mL of PFOA exposure from a meta-analysis of two studies (Bartell and Vieira 2021). The same methodology was applied as with kidney cancer to determine ORs, convert to RRs using a prevalence of 0.0817% of adult males, and identify a weighted AF (US Cancer Statistics Working Group 2020). The AF was then multiplied by the US population of adult males and baseline incidence of testicular cancer of 5.7 per 100,000 to identify the PFOA-attributable cases of testicular cancer in 2018 (Table S4) (Surveillance Research Program Surveillance, Epidemiology, and End Results Program). The number of PFOA-attributable testicular cancer cases was multiplied by $26,236, the estimated cost of each new case to the US healthcare system in 2018 dollars (Aberger et al. 2014). As with kidney cancer, we calculated the indirect 10-year cost of testicular cancer as lost DALY (0.288 for each year, valued at $50,000/year) over 10 years, discounting 3% per year for future preference, which we then summed with the direct cost of a new case of testicular cancer (Neumann et al. 2014).

### Hypothyroidism

Wen et al.’s analysis based on 2007–2010 NHANES data from 1181 adults provided us with an OR of 7.42 (95% CI 1.14–48.12) to estimate the increase in subclinical hypothyroidism per ln-unit increase of PFOA serum concentration (Wen et al. 2013). We conducted a sensitivity analysis using the association between PFOS and increased odds of subclinical hypothyroidism in females from the same study (OR 3.03; 95% CI 1.14–8.07).

For both PFAS, we applied the OR to our exposure percentiles of PFOA/PFOS in adult women then converted to an RR using a prevalence of clinical hypothyroidism of 0.3% (Hollowell et al. 2002). A weighted AF was then calculated and multiplied by the US population of adult women and incidence of subclinical hypothyroidism of 226.2 per 100,000 adults to obtain PFOA-attributable cases of subclinical hypothyroidism (Garmendia Madariaga et al. 2014). This was adjusted downward by 0.3% to account for the baseline prevalence of hypothyroidism (Table S5) (Hollowell et al. 2002).

The annual direct medical cost per case of hypothyroidism is valued at $2555 with associated $171 in indirect costs due to lost productivity in 2015 (Hepp et al. 2021). We converted the sum of these costs into 2018 dollars ($2888) and multiplied by the PFAS-attributable cases for a total annual cost of subclinical hypothyroidism in adult females. Given the variable clinical course of hypothyroidism, we chose to calculate costs for a single year of treatment due to PFAS rather than lifelong costs. As hypothyroidism is a chronic disease, we modeled an indirect 10-year cost as lost DALY (0.019 for each year, valued at $50,000/year) over 10 years, discounting 3% per year for future preference (Neumann et al. 2014).

### Adult Obesity

To quantify PFOS-attributable adult obesity, we modeled increases in obesity by shifting the mean BMI for US adults age > 18 years in relation to PFOS exposure in each centile and estimated increases in percentages of the population with BMI > 30 kg/m^2^. We applied results from Liu et al.’s study of 520 adults followed for 6–24 months after the cessation of a 2-year clinical trial of energy-restricted diets on weight change that reported those with PFOS levels > 32.1 ng/mL gained 3.2 kg over the 6–24-month study period, those with levels 19.2–32.1 ng/mL gained 3.5 kg, and those with levels < 19.2 ng/mL gained 1.5 kg (Liu et al. 2018a). Weight gain across tertiles was then linearized across the percentiles to estimate a finer distinction between those with varying exposures (Table S6, Table S14, Fig. S1).

After applying NHANES 2017–2018 PFOS levels to calculate attributable annual weight gain, the additional weight was added to mean weight in the unexposed scenario, as calculated from mean BMI (29.78 kg/m^2^) and height (1.66 m) and an exposed mean BMI was calculated from the new weight and same height. Increases in obesity (BMI > 30 kg/m^2^) in each exposed subpopulation were calculated by subtracting the percent obese in the exposed scenario to the unexposed counterfactual. The increase in obesity was multiplied by the annual number of adults in the US in 2018 as estimated by the US Census and adjusted for a baseline prevalence of obesity (42.4%) to obtain the number of cases of incident obesity among adults over the age of 18 attributable to PFOS (Table S6) (Fryar et al. 2016; Hales et al. 2020).

We estimated the long-term cost of obesity as a sum of the 15-year direct annual medical cost of obesity (e.g., medical expenses) and the indirect cost of quality-adjusted life years (QALY) lost, using a single age group as a model. We selected 35-year olds, as obesity rates increase with age and this age cohort would allow us to model a 15-year period with the assumption that the majority of 35-year-old obese individuals will remain obese and continue to live for at least 15 years. Using the annual direct medical cost of adult obesity as $2741 in 2005 dollars and discounting for future preference (3% annually), we calculated that a 35-year old who became obese as a result of PFAS exposure in 2017–2018 would incur $43,334 in direct medical costs over 15 years (Cawley and Meyerhoefer 2012). This cost was multiplied by the incremental increase in obesity and the total population of 35-year olds in the US. The indirect cost of adult obesity due to PFAS was calculated as QALY lost due to obesity, with each QALY assigned a value of $50,000 (Eq. 4) (Muennig et al. 2006; Neumann et al. 2014). Results for males and females were calculated separately, as QALY lost to obesity are sex specific (4.4 years for men and 7.2 years for women), and the final indirect costs for each PFAS of both genders were summed (Muennig et al. 2006).

4 $$ {\text{Indirect}}\, {\text{cost}} = \frac{{\$ 50,000 \times {\text{population}} {\text{obese}} \times {\text{QALY}}}}{{\left( {1.03^{15} } \right)}}. $$

### Type 2 Diabetes

We extrapolated incident cases of T2D in 2017–2018 due to PFOA exposure in females over age 18 years using the findings of a case–control study of 1586 women nested within the Nurses’ Health Study II that found higher odds of T2D associated with each tertile increase in PFOA concentration (Sun et al. 2018). Odds of incident T2D were linearized across tertiles to estimate a finer distinction with varying exposures as described for adult obesity. We converted the ORs to RRs and then applied Levin’s equation to calculate AFs from the RRs (Levin 1953). For each exposed subpopulation, the calculated AF was multiplied by the incidence rate of T2D [6.9 per 1000 American adults (CDC 2020)] and the annual population of US women in 2018. To avoid overestimation, we adjusted for baseline prevalence of T2D (13.0%) to obtain a final estimate of PFOA-attributable cases of T2D in adult women (Table S7) (CDC 2020). The lifetime cost of T2D was estimated at $93,183 per individual in 2018 and multiplied by the number of PFOA-attributable cases in 2018 (Zhou et al. 2013).

### Gestational Diabetes

We applied findings from a prospective cohort study of 501 women in whom preconception serum PFOA levels were associated with GDM (OR 1.61 per 0.43 SD increase in PFOA concentration; 95% CI 1.14–3.02) (Zhang et al. 2015). We assumed levels of PFOA exposure among women age 18–49 years in 2017–2018 NHANES to be similar to those in pregnant women of the same year and applied a reference level of 0.1 ng/mL below which we assumed no effect. As with prior calculations, we converted ORs to RRs and then applied Levin’s equation to calculate AFs from the RRs (Levin 1953). The AF across all centiles was multiplied by the number of births in 2017–2018 and the prevalence rate of GDM (5.60%) to estimate the annual PFOA-attributable cases of incident GDM (assuming that the prevalence of GDM is the same as the incidence, as the natural progression of the disease is < 1 year) (Table S8) (CDC/NCHS 2014).

Each case of GDM was estimated to have an annual medical cost of $12,089 and lifetime cost due to lost productivity for adverse birth outcomes associated with GDM of $56,237 in 2018 dollars (Peterson et al. 2015). These costs were multiplied by the number of PFAS-attributable cases of GDM.

### Endometriosis

After determining the percentile groupings of serum PFOA levels in women age 18–49, we utilized ORs for associations between PFOA and endometriosis from the Endometriosis: Natural History, Diagnosis, and Outcomes (ENDO) study, a case–control study of 495 women age 18–44 years that found an association between serum PFOA levels and higher odds of endometriosis (Buck Louis et al. 2012). We calculated ORs for PFOA in each percentile grouping based upon exposure levels in NHANES 2017–2018, applying a reference level of 0.1 ng/mL below which we assumed there was no increase in odds of endometriosis. We converted the ORs to RRs and applied Levin’s equation to calculate AFs as with prior outcomes (Levin 1953). The population incidence of endometriosis, 237 per 100,000 women/year, was obtained from the Nurses’ Health Study and multiplied by the AF across the modeled range of population exposures and the US Census population estimates of the number of women age 18–49 years in 2018 and then adjusted for baseline prevalence (6.1%) (Fuldeore and Soliman 2017) to quantify incident cases of endometriosis attributable to PFOA exposure (Table S9) (Missmer et al. 2004).

Following the methodology of Attina et al. (2016), we modeled the direct cost of endometriosis as the total healthcare costs over 10 years of treatment, valued at $30,292 in 2018 dollars (Fuldeore et al. 2015). We also calculated the indirect cost of endometriosis by aggregating lost DALY (0.123 for each year with endometriosis, valued at $50,000/year) over 10 years, discounting 3% per year for future preference (Neumann et al. 2014). These costs were multiplied by the newly incident cases of endometriosis attributable to annual PFOA exposure to obtain the annual PFOA-attributable economic burden.

### Polycystic Ovarian Syndrome

We quantified incident cases of PCOS in women age 15–45 years attributable to PFOA/PFOS by applying ERRs from a case–control study by Vagi et al. of 52 PCOS patients and 50 controls in Los Angeles to our percentile groupings of PFOA and PFOS exposure (Vagi et al. 2014). Linearized ORs were calculated as described for adult obesity, and ORs for exposure percentile groups were assigned based on the corresponding tertiles of exposure identified in the Vagi et al. study. The ORs for the second and third tertiles versus the first were 1.65 and 6.93 for PFOA (*p*_trend_ = 0.003) and 3.43 and 5.79 for PFOS (*p*_trend_ = 0.005), respectively (Vagi et al. 2014). ORs were converted to RRs, which were then converted to AFs using Levin’s equation, (Levin 1953) multiplied by the incidence of PCOS (2 per 1000 women based on a study of PCOS incidence in the United Kingdom) (Ding 2017) and the population of women age 15–45 years in the US (USCBureau 2020), and adjusted for baseline cases of PCOS (6.6%) (Azziz et al. 2004) to calculate the number of PFOA/PFOS-attributable cases of PCOS in 2018 (Table S10).

The annual medical cost of PCOS in the US was estimated as $4.37 billion for 4 million women or $1092 per PCOS case in 2004 dollars (Azziz et al. 2005). This cost estimate includes the annual cost of initial evaluation and treatment of associated menstrual dysfunction, infertility, T2D, and hirsutism. The cost per case was then multiplied by the PFOA/PFOS-attributable cases of PCOS and adjusted to 2018 dollars ($1452 per case) to determine the annual economic burden due to PCOS-related healthcare visits.

### Couple Infertility

We quantified PFAS-attributable cases of couple infertility, defined as TTP > 12 months, based on exposure data from 2017 to 2018 NHANES in women of childbearing age (age 18–49 years). To calculate the OR for infertility in each exposure group, we leveraged data from a case–control analysis of 910 women nested within the Norwegian Mother and Child Cohort Study (Whitworth et al. 2012). Although TTP is a couple-based outcome, chemical exposures were measured only in women, a common limitation among TTP studies. We calculated a linearized OR for estimated serum PFOA/PFOS in each exposure group and assigned an OR for infertility based on the corresponding quartile from the Norwegian study. As with prior estimates, we converted the OR to a RR based on a prevalence rate of impaired fecundity (13.1%) (CDC 2018) and subsequently calculated AFs using Levin’s equation (CDC 2018; Levin 1953). We multiplied the AFs by the incidence of infertility in 2018 (63.6 per 10,000 women) and the US population of women age 18–49 years and then adjusted for the baseline prevalence of infertility (13.1%) to quantify attributable cases of infertility (Table S11) (Boivin et al. 2007; Stahlman and Fan 2019).

We applied a 56% utilization rate of assisted reproductive technologies (ART) among infertile couples to assess cost (Boivin et al. 2007). The cost of a single fresh cycle of ART was valued at $63,530 in 2018 dollars. This cost is inclusive of direct maternal and infant costs from 27 weeks prior to delivery through the first year of an infant’s life and accounts for the increased rate of multiparity and premature births associated with ART (Crawford et al. 2016). This cost was multiplied by the PFOA/PFOS-attributable annual use of ART in 2018 to estimate the total cost.

### Breast Cancer

We applied an OR of 1.26 per ng/mL of PFOA and 1.02 per ng/mL of PFOS from a case–control study of 161 Inuit women in Greenland (Wielsoe et al. 2017). We then calculated the ORs for PFOA/PFOS-associated breast cancer for each of our exposure centiles by multiplying the ORs from this study by the levels of exposure from 2017 to 2018 NHANES among women age 18–49, assuming a reference level of 0.1 ng/mL below which we modeled no effect. The OR for each centile was converted to an RR, which was further transformed into an AF. PFOA/PFOS-attributable cases of breast cancer were then determined by multiplying the weighted AFs by the population of women age 18–49 years and the US breast cancer incidence rate (125.1 per 100,000) and then adjusting for a baseline prevalence of 1.2% (Table S12) (US Cancer Statistics Working Group 2020).

The PFOA/PFOS-attributable cases of female breast cancer were multiplied by the healthcare costs for the first 6 months of a new breast cancer diagnosis. While there are varied lifetime cost estimates of having breast cancer depending on the different stages at which patients are diagnosed, $51,498 in 2018 dollars is the minimum estimated cost a patient will incur throughout the first 6 months of diagnosis regardless of prognosis or odds of remission (Lamerato et al. 2006). As with kidney and testicular cancer, we calculated the indirect 10-year cost of breast cancer as lost DALY (0.288 for each year, valued at $50,000/year) over 10 years, discounting 3% per year for future preference (Neumann et al. 2014).

### Pneumonia

To determine the PFAS-attributable increase in pneumonia infections among children age < 3 years, we utilized RRs of 1.27 (95% CI 1.12–1.43) and 1.20 (95% CI 1.07–1.34) for PFOA and PFOS, respectively, from an analysis of 1270 maternal-child pairs in the Norwegian Mother and Child Cohort Study (Impinen et al. 2019). We applied the RR to each percentile grouping of maternal serum PFOA/PFOS levels to calculate the increased risk of pneumonia and bronchitis among children age < 3 years as a result of in utero exposure to PFAS. The RR was then transformed to a weighted AF across all centiles using Levin’s equation (Levin 1953) and multiplied by the US population of children age < 3 years and a weighted average incidence rate of 49.4 per 10,000 children, as derived from the incidence rates of pneumonia in children < 2 years old and 2–4 years old, to obtain the PFOA/PFOS-attributable cases of pneumonia in children under age 3 (Table S13) (Jain et al. 2015).

The economic burden of pneumonia in children age < 3 years was constructed as a combination of the overall direct cost of a pneumonia episode (emergency room visit, hospitalization, or outpatient treatment) and the indirect cost defined as lost parental weekly earnings. The average cost across all healthcare settings per case of pneumonia was determined to be $2952 in 2018 dollars (Tong et al. 2018). For the indirect cost, we multiplied the mean weekly earnings of full-time wage and salary workers in 2014 ($113 per diem) by the average length of stay for a pneumonia hospitalization (3.1 days) to obtain an indirect cost of $350 in lost parental earnings per case or $372 in 2018 dollars (US Bureau of Labor Statistics 2021; Williams et al. 2018). We multiplied both costs by the number of PFOA/PFOS-attributable cases to identify total direct and indirect costs.

## Results

Risk-of-bias tools yielded consistent evaluations of the quality of articles used as sources for ERRs across the two reviewers. Among the systematic reviews, the Steenland et al. review of associations with LBW was identified as having low risk of bias except for its reliance on a single source for studies (PubMed), failure to identify whether a single author or multiple authors assessed studies for inclusion and extracted data, and lack of risk-of-bias analysis. The Liu et al. review of childhood obesity and Kim et al. review of hypothyroidism were both identified to have low risk across all four domains by both authors. The Bartell et al. meta-analysis was based on data from articles identified in a prior review (Steenland and Winquist 2021) that also relied exclusively on PubMed, failed to identify who reviewed studies for inclusion and extracted data, and lacked a risk-of-bias analysis. Also, their calculations were based on data from only four studies of kidney cancer and two studies of testicular cancer. All four reviews appropriately considered the relevance of the identified research studies to the questions being considered and avoided emphasis on statistical significance, while two of the systematic reviews (Steenland and Bartell) were identified as probably (vs. conclusively) having addressed all concerns in the four domains. The overall risk of bias was identified as low for all four systematic reviews used (Table S15).

Both reviewers evaluated studies as definitely low risk of bias across all domains for subclinical hypothyroidism, T2D in females, childhood obesity, endometriosis, PCOS, and female breast cancer. The Meng et al. study of LBW, Zhang et al. study of GDM, and Impinem et al. study of pneumonia in children were judged by both reviewers to have potential live birth bias as a threat to internal validity, reducing the corresponding domain’s assessment to probably low risk of bias. Both reviewers identified the Whitworth et al. study of couple infertility as potentially having conception bias, yielding a probably low risk of bias for internal validity, as well. One reviewer (LK) noted that the Liu et al. study of adult obesity used data collected as part of a randomized controlled trial, meaning the results may not be generalizable, and therefore evaluated the study as probably low risk of bias for internal validity. All of the evaluated studies at minimum had probably low risk of bias across all criteria, as evaluated by both reviewers (Table S16).

We identified PFOA-attributable disease costs in the US in 2018 of $5.52 billion across the five primary disease endpoints based on meta-analytic ERRs. This estimate represented the lower bound of possible costs, with our sensitivity analyses revealing as much as $62.6 billion in overall costs of long-chain PFAS exposure. Attributable fractions for PFAS of disease burden ranged from 0.08% for testicular cancer due to PFOA to 30.7% for LBW due to PFOS (Table 4).

The largest economic contributor to the main estimate of disease costs attributable to PFAS was childhood obesity ($2.65 billion). For childhood obesity, we also modeled a total lifetime direct medical cost of $4.56 billion due to PFOS exposure, which represents the incremental lifetime medical costs of a child becoming obese at age 10 relative to a child with normal BMI. Hypothyroidism in females contributed $1.26 billion in annual cost. This is a composite value of $42.1 million in direct costs of new cases of hypothyroidism and $1.22 billion in indirect costs as DALY lost over 10 years. The total PFOS-attributable cost for hypothyroidism in the sensitivity analysis was $5.18 billion. PFOA-attributable kidney and testicular cancer contributed a total of $4.88 million in direct costs and $187 million in indirect costs as DALY lost over 10 years. LBW due to PFOA exposure added $1.42 billion in healthcare expenditures annually; this estimate was a composite of $305 million due to direct costs of hospitalization associated with a LBW newborn and $1.11 billion attributable to lost IQ points associated with LBW. The cost estimates for PFOS exposure were substantially higher: $2.94 billion due to hospitalization costs and $10.7 billion due to lost IQ.

The highest costs we identified in both the main and sensitivity analyses were PFOS-attributable lifetime costs related to adult obesity, totaling $17.0 billion dollars annually. We estimated the PFOS-associated 15-year direct medical cost of obesity in newly obese 35-year olds as $3.21 billion, with $13.8 billion in QALY lost over the same 15 years. Other metabolic outcomes included T2D, for which the lifetime cost of PFOA-attributable annual incident cases in women was $140 million, and GDM, for which the low estimate totaled $414 million in annual costs due to PFOA exposure: $73.3 million in direct medical costs and $341 million in indirect costs of lost productivity secondary to adverse birth effects of GDM.

Women’s gynecologic and reproductive health outcomes were also major contributors to the total calculated for the sensitivity analysis. Annual incident PFOA-attributable cases of endometriosis accounted for $397 million to $10.2 billion in total costs, with $21.1 to $547 million due to direct medical costs over 10 years and $376 million to $9.76 billion due to DALY lost over the same 10 years. The annual cost of initial evaluation of PCOS and treatment of associated menstrual dysfunction, infertility, T2D, and hirsutism generated at least $10.5 million PFOA-attributable cost estimate, with a higher estimate of $10.9 million. We estimated the cost of PFOA-attributable cases of couples seeking ART per annum to be at minimum $37.6 million ($1.66 billion upper bound) based on the price of a single embryo transfer ART cycle and the increase in medical costs associated with increased multiparity because of ART. For breast cancer due to PFOA exposure, we estimated $159 million in direct medical cost of utilization of healthcare services within the first 6 months of a new breast cancer diagnosis and $3.92 billion in DALY lost over 10 years. Finally, we estimated PFOA-attributable pneumonia in children < 3 years of age to cost the US medical system $1.49 to $22.5 billion annually due to treatment costs and indirect costs of parental absenteeism.

## Discussion

PFAS contribute substantially to disease and disability in the US, with at least $5.52 billion and as much as $62.6 billion in associated economic costs. Our study builds on prior papers that have examined the disease burden and costs associated with PFAS exposure by incorporating 13 health outcomes for which evidence is strongest and constructing a range of models to estimate disease burden and economic costs. The findings suggest that the cost of remediation and of substituting PFAS with safer alternatives in consumer products may well be justified by the large economic costs of adverse health outcomes associated with PFAS exposure.

These estimates are highly conservative for multiple reasons. We did not include outcomes reported by the C8 Science Panel that were not confirmed in general population studies, as those associations were identified in a highly exposed population and our focus was on estimating the disease burden and economic costs due to routine exposure. We also did not include endpoints for which not enough consistent evidence has accumulated, such as prematurity, attention-deficit hyperactivity disorder, and lowered IQ in children resulting from prenatal exposure, and prostate cancer in adult men (Kahn et al. 2020). We based our minimum estimate on the costs associated with a single PFAS (PFOA) for each exposure-disease association and did not aggregate costs across multiple members of the PFAS class, when evidence suggests additivity and synergy in this class of > 4700 chemicals (Chohan et al. 2020). We quantified disease burden for only those associations with strongest scientific evidence for probable causation. We aggregated published costs for each of the diseases considered, but our calculations do not capture the real and substantial social costs such as pain and suffering to patients with PFAS-attributable conditions and effects on their loved ones (Cordner et al. 2021).

Our approach has several limitations. Our analysis relies on previously conducted studies to provide ERRs between PFOA/PFOS exposure and the outcomes of interest. These studies may not be generalizable to the current US population due to recent shifts away from the use of PFOA and PFOS in manufacturing; indeed, median serum levels of PFOA and PFOS in the US have declined substantially from 2007–2008 to 2017–2018, although production of—and consequent human exposure to—replacement PFAS, such as GenX, which are at least as toxic as PFOA (United States Environmental Protection Agency 2021), have increased. Despite the vast literature that exists on the endocrine-disrupting effects of PFAS, there have yet to be large cohort studies to evaluate the longitudinal effects of PFAS exposure in humans and decades of epidemiologic data are required before causation may be acknowledged and attributable disease burden calculated with more certainty. However, the risk-of-bias assessments yielded probably to definitely low risk of bias, and the stakes of inaction are high enough to justify action. It is also important to note that there is likely an overlap in some of the indirect costs modeled in our analysis due to high rates of comorbidity of endocrinopathies, e.g., the indirect lifetime costs of adult obesity may overlap with the costs of T2D.

Despite the limitations of our analysis, our models provide an approximation of the scope of the disease burden and associated costs attributable to exposure to these ubiquitous chemicals. As more research investigates the endocrine-disrupting effects of other chemicals in the PFAS class currently prevalent in manufacturing processes, it is likely that the PFAS-attributable disease burden and associated costs will continue to increase, further strengthening the case for regulation of the entire class of chemicals. Further action is urgently needed to limit these exposures from a health equity perspective, as exposure to these chemicals is not distributed equally throughout the US population and there are subsets who bear more of a burden, e.g., those who live near airports, military installations, and industrial plants (Attina et al. 2019).

## Conclusion

The present study identifies at least $5.52 billion in annual disease burden and associated social costs of current annual exposure to long-chain PFAS with our sensitivity analyses revealing as much as $62.6 billion. Regulatory action to limit ongoing PFAS use and remediate contaminated water supplies may produce substantial economic benefits.

## Supplementary Information

Below is the link to the electronic supplementary material.

Supplementary file1 (DOCX 67 KB)

## Data Availability

Data relating to this publication will be provided upon reasonable request.

## Abbreviations

AF: Attributable fraction
ART: Assisted reproductive technology
BMI: Body mass index
CI: Confidence interval
DALY: Disability-adjusted life years
ERR: Exposure–response relationship
GDM: Gestational diabetes
IQ: Intelligence quotient
LBW: Low birth weight
NHANES: National Health and Nutrition Examination Survey
OR: Odds ratio
PCOS: Polycystic ovarian syndrome
PFAS: Perfluoroalkyl substances
PFOA: Perfluorooctanoic acid
PFOS: Perfluorooctane sulfonic acid
ppt: Parts per trillion
QALY: Quality-adjusted life years
RR: Risk ratio
SD: Standard deviation
TTP: Time to pregnancy
T2D: Type 2 diabetes mellitus
US: United States
