# Supplementary material for: Leveraging Systematic Reviews to Explore Disease Burden and Costs of Per- and Polyfluoroalkyl Substance Exposures in the United States
Source: Expo Health. 2022 Jul 26;15(2):373–94. doi: 10.1007/s12403-022-00496-y (PMC10198842; doi:10.1007/s12403-022-00496-y)
Supplement: Supplementary file 1 — Supplementary file1 (DOCX 67 KB) [file 12403_2022_496_MOESM1_ESM.docx]

| **Table S1. Low Birth Weight** | | | | | | | | |
| --- | --- | --- | --- | --- | --- | --- | --- | --- |
| Exposure | Percentile range of exposure | 0-9 | 10-24 | 25-49 | 50-74 | 75-89 | 90-99 | >99 |
| PFOA | Assigned exposure concentration (ng/mL) | 0.00 | 0.47 | 0.67 | 0.97 | 1.47 | 2.37 | 5.17 |
|  | Threshold of exposure | 0.1 ng/mL | | | | | | |
|  | Attributable decrease in birth weight (grams) | 0.00 | 3.89 | 5.99 | 9.14 | 14.4 | 23.8 | 53.2 |
|  | Low birth weight births attributable to PFOA | 0 | 22 | 923 | 1764 | 2427 | 1997 | 2919 |
|  | Total attributable cases of low birth weights | 10053 | | | | | | |
|  | Direct cost of hospitalization | $305,242,373 | | | | | | |
|  | Indirect cost due to IQ lost | $1,110,864,673 | | | | | | |
| PFOS~ | Assigned exposure concentration (ng/mL) | 0.00 | 1.10 | 1.70 | 2.60 | 3.90 | 5.70 | 11.9 |
|  | Threshold of exposure | 0.1 ng/mL | | | | | | |
|  | Attributable decrease in birth weight (grams) | 0.00 | 156 | 185 | 212 | 239 | 264 | 312 |
|  | Low birth weight births attributable to PFOS | 0 | 6807 | 12732 | 23663 | 25799 | 16132 | 12092 |
|  | Averaged attributable cases of low birth weights | 97226 | | | | | | |
|  | Direct cost of hospitalization | $2,952,188,214 | | | | | | |
|  | Indirect cost due to IQ lost | $10,743,860,889 | | | | | | |
|  | Direct cost of hospitalization per LBW birth* | $30,364 | | | | | | |
|  | Indirect cost due to lost IQ point* | $22,190 | | | | | | |
| * adjusted to 2018 dollars | |  |  |  |  |  |  |  |
| ~ used for high cost estimate in sensitivity analysis | |  |  |  |  |  |  |  |
|  |  |  |  |  |  |  |  |  |

|  |  |  |  |  |  |  |  |  |
| --- | --- | --- | --- | --- | --- | --- | --- | --- |
| **Table S2. Childhood Obesity at Age 10** | | | | | | | | |
| Exposure | Percentile range of exposure | 0-9 | 10-24 | 25-49 | 50-74 | 75-89 | 90-99 | >99 |
| PFOA | Assigned exposure concentration (ng/mL) | 0.00 | 2.30 | 2.70 | 3.50 | 4.60 | 5.90 | 7.10 |
|  | Threshold of exposure | 0.1 ng/mL | | | | | | |
|  | Incremental increase in z-score | 0.00 | 0.20 | 0.23 | 0.31 | 0.40 | 0.52 | 0.63 |
|  | Incremental frequency of increase in BMI z-score | 0.00 | 0.02 | 0.03 | 0.04 | 0.06 | 0.08 | 0.10 |
|  | Attributable fraction of increase in BMI-age-for-sex z-score | 3.78% | | | | | | |
|  | Attributable cases of obesity at age 10 | 127362 | | | | | | |
|  | Lifetime cost of attributable obesity at age 10 | $2,646,628,268 | | | | | | |
| PFOS~ | Assigned exposure concentration (ng/mL) | 0.00 | 4.70 | 7.20 | 9.90 | 17.7 | 24.3 | 32.2 |
|  | Threshold of exposure | 0.1 ng/mL | | | | | | |
|  | Incremental increase in z-score | 0.00 | 0.68 | 0.75 | 0.81 | 0.91 | 0.97 | 1.02 |
|  | Incremental frequency of increase in BMI z-score | 0.00 | 0.12 | 0.14 | 0.15 | 0.18 | 0.20 | 0.22 |
|  | Attributable fraction of increase in BMI-age-for-sex z-score | 13.70% | | | | | | |
|  | Attributable cases of obesity at age 10 | 462119 | | | | | | |
|  | Lifetime cost of attributable obesity at age 10 | $9,602,971,982 | | | | | | |
|  | Lifetime cost of obesity at age 10 per case* | $20,780 | | | | | | |
| * adjusted to 2018 dollars | |  |  |  |  |  |  |  |
| ~ used for high cost estimate in sensitivity analysis | |  |  |  |  |  |  |  |

|  |  |  |  |  |  |  |  |  |
| --- | --- | --- | --- | --- | --- | --- | --- | --- |
| **Table S3. Kidney Cancer** | | | | | | | | |
| Exposure | Percentile range of exposure | 0-9 | 10-24 | 25-49 | 50-74 | 75-89 | 90-99 | >99 |
| PFOA | Assigned exposure concentration (ng/mL) | 0.00 | 0.67 | 0.97 | 1.47 | 2.17 | 3.07 | 8.30 |
|  | Threshold of exposure | 0.1 ng/mL | | | | | | |
|  | OR of kidney cancer | 1.00 | 1.01 | 1.01 | 1.02 | 1.03 | 1.05 | 1.13 |
|  | Relative risk of kidney cancer | 1.00 | 1.01 | 1.01 | 1.02 | 1.03 | 1.05 | 1.13 |
|  | Incremental increase of kidney cancer | 0.00 | 0.00 | 0.00 | 0.01 | 0.00 | 0.00 | 0.00 |
|  | Attributable fraction of kidney cancer | 0.33% | | | | | | |
|  | Attributable cases of kidney cancer | 142 | | | | | | |
|  | Cost of first year after kidney cancer diagnosis | $4,742,215 | | | | | | |
|  | Indirect cost of attributable kidney cancer as DALY lost over 10 years | $179,180,181 | | | | | | |
|  | Cost per case of first year after kidney cancer diagnosis* | $33,485 | | | | | | |
| * adjusted to 2018 dollars | |  |  |  |  |  |  |  |
|  |  |  |  |  |  |  |  |  |
| **Table S4. Testicular Cancer** | | | | | | | | |
| Exposure | Percentile range of exposure | 0-9 | 10-24 | 25-49 | 50-74 | 75-89 | 90-99 | >99 |
| PFOA | Assigned exposure concentration (ng/mL) | 0.00 | 0.87 | 1.17 | 1.67 | 2.27 | 3.27 | 8.30 |
|  | Threshold of exposure | 0.1 ng/mL | | | | | | |
|  | OR of testicular cancer | 1.00 | 1.00 | 1.00 | 1.00 | 1.01 | 1.01 | 1.02 |
|  | Relative risk of testicular cancer | 1.00 | 1.00 | 1.00 | 1.00 | 1.01 | 1.01 | 1.02 |
|  | Incremental increase of testicular cancer | 0.00 | 0.00 | 0.00 | 0.00 | 0.00 | 0.00 | 0.00 |
|  | Attributable fraction of testicular cancer | 0.08% | | | | | | |
|  | Attributable cases of testicular cancer | 5 | | | | | | |
|  | Cost of first year after testicular cancer diagnosis | $139,073 | | | | | | |
|  | Indirect cost of attributable testicular cancer as DALY lost over 10 years | $6,706,561 | | | | | | |
|  | Cost per case of first year after testicular cancer diagnosis* | $26,236 | | | | | | |
| * adjusted to 2018 dollars | |  |  |  |  |  |  |  |

|  |  |  |  |  |  |  |  |  |
| --- | --- | --- | --- | --- | --- | --- | --- | --- |
| **Table S5. Hypothyroidism in Females** | | | | | | | | |
| Exposure | Percentile range of exposure | 0-9 | 10-24 | 25-49 | 50-74 | 75-89 | 90-99 | >99 |
| PFOA | Assigned exposure concentration (ng/mL) | 0.00 | 0.47 | 0.67 | 0.97 | 1.47 | 2.37 | 5.17 |
|  | Threshold of exposure | Same outcome for 0.1 ng/mL and 1 ng/mL | | | | | | |
|  | Odds ratio of hypothyroidism in females | 1.00 | 1.00 | 1.00 | 1.00 | 2.16 | 5.64 | 26.9 |
|  | Relative risk of hypothyroidism in females | 1.00 | 1.00 | 1.00 | 1.00 | 2.16 | 5.56 | 25.0 |
|  | Incremental increase of hypothyroidism in females | 0.00 | 0.00 | 0.00 | 0.00 | 0.15 | 0.29 | 0.19 |
|  | Attributable fraction of hypothyroidism in females | 5.03% | | | | | | |
|  | Attributable cases of hypothyroidism in females | 14572 | | | | | | |
|  | Cost of hypothyroidism in females | $42,083,427 | | | | | | |
|  | Indirect cost of attributable hypothyroidism as DALY lost over 10 years | $1,216,259,125 | | | | | | |
| PFOS~ | Assigned exposure concentration (ng/mL) | 0.00 | 1.10 | 1.70 | 2.60 | 3.90 | 5.70 | 11.9 |
|  | Threshold of exposure | Same outcome for 0.1 ng/mL and 1 ng/mL | | | | | | |
|  | Odds ratio of hypothyroidism in females | 1.00 | 1.11 | 1.80 | 2.88 | 4.52 | 6.89 | 15.6 |
|  | Relative risk of hypothyroidism in females | 1.00 | 1.11 | 1.80 | 2.87 | 4.47 | 6.77 | 14.9 |
|  | Incremental increase of hypothyroidism in females | 0.00 | 0.02 | 0.17 | 0.32 | 0.34 | 0.34 | 0.12 |
|  | Attributable fraction of hypothyroidism in females | 20.69% | | | | | | |
|  | Attributable cases of hypothyroidism in females | 59939 | | | | | | |
|  | Cost of hypothyroidism in females | $173,106,006 | | | | | | |
|  | Indirect cost of attributable hypothyroidism as DALY lost over 10 years | $5,002,961,377 | | | | | | |
|  | Cost of hypothyroism per case* | $2,888 | | | | | | |
| * adjusted to 2018 dollars | |  |  |  |  |  |  |  |
| ~ used for high cost estimate in sensitivity analysis | |  |  |  |  |  |  |  |

|  |  |  |  |  |  |  |  |  |
| --- | --- | --- | --- | --- | --- | --- | --- | --- |
| **Table S6. Adult Obesity** | | | | | | | | |
| Exposure | Percentile range of exposure | 0-9 | 10-24 | 25-49 | 50-74 | 75-89 | 90-99 | >99 |
| PFOS | Assigned exposure concentration (ng/mL) | 0.00 | 1.60 | 2.70 | 4.70 | 7.80 | 12.0 | 26.2 |
|  | Threshold of exposure | Same outcome for 0.1 ng/mL and 1 ng/mL | | | | | | |
|  | Kilograms gained based on tertile of exposure | 1.50 | 1.50 | 1.50 | 1.50 | 1.50 | 1.50 | 3.50 |
|  | Linearized kilograms gained | 1.26 | 1.36 | 1.48 | 1.67 | 1.94 | 2.63 | 4.31 |
|  | Incremental increase in obesity | 0.02 | 0.03 | 0.03 | 0.03 | 0.04 | 0.05 | 0.08 |
|  | Attributable fraction of obesity | 2.98% | | | | | | |
|  | Attributable cases of obesity in all adults | 4294379 | | | | | | |
|  | Direct medical costs of attributable obesity at age 35 | $3,213,478,888 | | | | | | |
|  | Indirect cost of QALY lost to obesity at age 35 | $13,793,387,068 | | | | | | |
|  | Direct cost of 15 year obesity per case* | $43,334 | | | | | | |
| * adjusted to 2018 dollars | |  |  |  |  |  |  |  |
|  |  |  |  |  |  |  |  |  |
| **Table S7. Adult-onset Type II Diabetes in Females** | | | | | | | | |
| Exposure | Percentile range of exposure | 0-9 | 10-24 | 25-49 | 50-74 | 75-89 | 90-99 | >99 |
| PFOA | Assigned exposure concentration (ng/mL) | 0.00 | 0.47 | 0.67 | 0.97 | 1.47 | 2.37 | 5.17 |
|  | Threshold of exposure | Same outcome for 0.1 ng/mL and 1 ng/mL | | | | | | |
|  | OR based on tertile of exposure | 1.00 | 1.00 | 1.00 | 1.00 | 1.00 | 1.00 | 1.27 |
|  | Linearized OR of diabetes | 1.00 | 1.00 | 1.00 | 1.04 | 1.09 | 1.30 | 1.99 |
|  | Relative risk of diabetes | 1.00 | 1.00 | 1.00 | 1.02 | 1.06 | 1.15 | 1.39 |
|  | Incremental increase of diabetes | 0.00 | 0.00 | 0.00 | 0.00 | 0.01 | 0.01 | 0.00 |
|  | Attributable fraction of diabetes | 0.37% | | | | | | |
|  | Attributable cases of diabetes in adult females | 1728 | | | | | | |
|  | Lifetime cost of attributable diabetes in adult females | $140,066,324 | | | | | | |
|  | Cost per case of diabetes* | $93,183 | | | | | | |
| * adjusted to 2018 dollars | |  |  |  |  |  |  |  |

|  |  |  |  |  |  |  |  |  |
| --- | --- | --- | --- | --- | --- | --- | --- | --- |
| **Table S8. Gestational Diabetes** | | | | | | | | |
| Exposure | Percentile range of exposure | 0-9 | 10-24 | 25-49 | 50-74 | 75-89 | 90-99 | >99 |
| PFOA | Assigned exposure concentration (ng/mL) | 0.00 | 0.47 | 0.67 | 0.97 | 1.47 | 2.37 | 5.17 |
|  | Threshold of exposure | 1 ng/mL | | | | | | |
|  | OR of GDM | 1.00 | 1.00 | 1.15 | 1.20 | 1.28 | 1.41 | 1.95 |
|  | Relative risk of GDM | 1.00 | 1.00 | 1.14 | 1.19 | 1.26 | 1.38 | 1.85 |
|  | Incremental increase of GDM | 0.00 | 0.00 | 0.03 | 0.04 | 0.04 | 0.03 | 0.01 |
|  | Attributable fraction of GDM | 2.85% | | | | | | |
|  | Attributable cases of GDM | 6061 | | | | | | |
|  | Annual medical cost of attributable GDM | $73,274,481 | | | | | | |
|  | Indirect cost of lost productivity | $340,875,830 | | | | | | |
| PFOA~ | Assigned exposure concentration (ng/mL) | 0.00 | 0.47 | 0.67 | 0.97 | 1.47 | 2.37 | 5.17 |
|  | Threshold of exposure | 0.1 ng/mL | | | | | | |
|  | OR of GDM | 1.00 | 1.00 | 1.40 | 1.44 | 1.53 | 1.65 | 2.31 |
|  | Relative risk of GDM | 1.00 | 1.00 | 1.37 | 1.41 | 1.49 | 1.59 | 2.16 |
|  | Incremental increase of GDM | 0.00 | 0.00 | 0.08 | 0.09 | 0.07 | 0.05 | 0.01 |
|  | Attributable fraction of GDM | 5.87% | | | | | | |
|  | Attributable cases of GDM | 12474 | | | | | | |
|  | Annual medical cost of attributable GDM | $150,797,420 | | | | | | |
|  | Indirect cost of lost productivity | $701,515,656 | | | | | | |
|  | Annual medical cost per case of GDM* | $12,089 | | | | | | |
|  | Lifetime cost of lost productivity per case* | $56,237 | | | | | | |
| * adjusted to 2018 dollars | |  |  |  |  |  |  |  |
| ~ used for high cost estimate in sensitivity analysis | |  |  |  |  |  |  |  |

|  |  |  |  |  |  |  |  |  |
| --- | --- | --- | --- | --- | --- | --- | --- | --- |
| **Table S9. Endometriosis** | | | | | | | | |
| Exposure | Percentile range of exposure | 0-9 | 10-24 | 25-49 | 50-74 | 75-89 | 90-99 | >99 |
| PFOA | Assigned exposure concentration (ng/mL) | 0.00 | 0.47 | 0.67 | 0.97 | 1.47 | 2.37 | 5.17 |
|  | Threshold of exposure | 1 ng/mL | | | | | | |
|  | Odds ratio of endometriosis | 1.00 | 1.00 | 1.00 | 1.00 | 1.11 | 1.27 | 1.57 |
|  | Relative risk of endometriosis accounting for prevalence of 6.10% | 1.00 | 1.00 | 1.00 | 1.00 | 1.10 | 1.25 | 1.52 |
|  | Incremental increase of endometriosis | 0.00 | 0.00 | 0.00 | 0.00 | 0.02 | 0.02 | 0.01 |
|  | Attributable fraction of endometriosis | 0.43% | | | | | | |
|  | Attributable cases of endometriosis | 696 | | | | | | |
|  | Direct medical cost of attributable endometriosis over 10 years | $21,094,634 | | | | | | |
|  | Indirect cost of attributable endometriosis as DALY lost over 10 years | $376,283,014 | | | | | | |
| PFOA~ | Assigned exposure concentration (ng/mL) | 0.00 | 0.47 | 0.67 | 0.97 | 1.47 | 2.37 | 5.17 |
|  | Threshold of exposure | 0.1 ng/mL | | | | | | |
|  | Odds ratio of endometriosis | 1.00 | 1.53 | 1.69 | 1.87 | 2.10 | 2.40 | 2.98 |
|  | Relative risk of endometriosis accounting for prevalence of 6.10% | 1.00 | 1.49 | 1.62 | 1.78 | 1.97 | 2.21 | 2.66 |
|  | Incremental increase of endometriosis | 0.00 | 0.07 | 0.13 | 0.16 | 0.13 | 0.10 | 0.02 |
|  | Attributable fraction of endometriosis | 11.27% | | | | | | |
|  | Attributable cases of endometriosis | 18062 | | | | | | |
|  | Direct medical cost of attributable endometriosis over 10 years | $547,137,555 | | | | | | |
|  | Indirect cost of attributable endometriosis as DALY lost over 10 years | $9,759,760,027 | | | | | | |
|  | Direct cost per case of endometriosis over 10 years* | $30,292 | | | | | | |
| * adjusted to 2018 dollars | |  |  |  |  |  |  |  |
| ~ used for high cost estimate in sensitivity analysis | |  |  |  |  |  |  |  |

|  |  |  |  |  |  |  |  |  |
| --- | --- | --- | --- | --- | --- | --- | --- | --- |
| **Table S10. PCOS** | | | | | | | | |
| Exposure | Percentile range of exposure | 0-9 | 10-24 | 25-49 | 50-74 | 75-89 | 90-99 | >99 |
| PFOA~ | Assigned exposure concentration (ng/mL) | 0.00 | 0.47 | 0.67 | 0.87 | 1.37 | 2.07 | 5.17 |
|  | Threshold of exposure | Same outcome for 0.1 ng/mL and 1 ng/mL | | | | | | |
|  | OR based on tertile of exposure | 1.00 | 1.00 | 1.00 | 1.00 | 1.00 | 1.00 | 6.93 |
|  | Linearized OR of PCOS | 1.00 | 1.00 | 1.03 | 1.44 | 2.16 | 4.42 | 10.9 |
|  | Relative risk of PCOS | 1.00 | 1.00 | 1.02 | 1.40 | 2.00 | 3.60 | 6.58 |
|  | Incremental increase of PCOS | 0.00 | 0.00 | 0.01 | 0.09 | 0.13 | 0.19 | 0.05 |
|  | Attributable fraction of PCOS | 6.16% | | | | | | |
|  | Attributable cases of PCOS | 7505 | | | | | | |
|  | Annual cost of attributable PCOS | $10,898,822 | | | | | | |
| PFOS | Assigned exposure concentration (ng/mL) | 0.00 | 1.10 | 1.70 | 2.50 | 3.60 | 5.30 | 10.7 |
|  | Threshold of exposure | Same outcome for 0.1 ng/mL and 1 ng/mL | | | | | | |
|  | OR based on tertile of exposure | 1.00 | 1.00 | 1.00 | 1.00 | 1.00 | 1.00 | 5.79 |
|  | Linearized OR of PCOS | 1.00 | 1.00 | 1.04 | 1.46 | 2.08 | 3.65 | 7.26 |
|  | Relative risk of PCOS | 1.00 | 1.00 | 1.04 | 1.42 | 1.94 | 3.11 | 5.14 |
|  | Incremental increase of PCOS | 0.00 | 0.00 | 0.01 | 0.09 | 0.12 | 0.16 | 0.04 |
|  | Attributable fraction of PCOS | 5.92% | | | | | | |
|  | Attributable cases of PCOS | 7209 | | | | | | |
|  | Annual cost of attributable PCOS | $10,469,348 | | | | | | |
|  | Cost per PCOS-related healthcare visit* | $1,452 | | | | | | |
| * adjusted to 2018 dollars | |  |  |  |  |  |  |  |
| ~ used for high cost estimate in sensitivity analysis | |  |  |  |  |  |  |  |

|  |  |  |  |  |  |  |  |  |
| --- | --- | --- | --- | --- | --- | --- | --- | --- |
| **Table S11. Couple Infertility** | | | | | | | | |
| Exposure | Percentile range of exposure | 0-9 | 10-24 | 25-49 | 50-74 | 75-89 | 90-99 | >99 |
| PFOA | Assigned exposure concentration (ng/mL) | 0.00 | 0.47 | 0.67 | 0.97 | 1.47 | 2.37 | 5.17 |
|  | Threshold of exposure | 1 ng/mL | | | | | | |
|  | OR of subfecundity | 1.00 | 1.00 | 1.00 | 1.00 | 1.07 | 1.16 | 1.33 |
|  | Relative risk of infertility | 1.00 | 1.00 | 1.00 | 1.00 | 1.06 | 1.14 | 1.28 |
|  | Incremental Increase of Infertility | 0.00 | 0.00 | 0.00 | 0.00 | 0.01 | 0.01 | 0.00 |
|  | Attributable fraction of Infertility | 0.25% | | | | | | |
|  | Attributable cases of ART utilized | 593 | | | | | | |
|  | Cost of attributable ART utilization | $37,649,064 | | | | | | |
| PFOS~ | Assigned exposure concentration (ng/mL) | 0.00 | 1.10 | 1.70 | 2.60 | 3.90 | 5.70 | 11.9 |
|  | Threshold of exposure | 0.1 ng/mL | | | | | | |
|  | OR of subfecundity | 1.00 | 1.65 | 1.80 | 1.97 | 2.14 | 2.31 | 2.70 |
|  | Relative risk of infertility | 1.00 | 1.52 | 1.63 | 1.75 | 1.86 | 1.97 | 2.21 |
|  | Incremental Increase of Infertility | 0.00 | 0.07 | 0.14 | 0.16 | 0.11 | 0.08 | 0.01 |
|  | Attributable fraction of Infertility | 10.86% | | | | | | |
|  | Attributable cases of ART utilized | 26160 | | | | | | |
|  | Cost of attributable ART utilization | $1,661,962,582 | | | | | | |
|  | Cost of cycle of ART and associated maternal/infant medical costs* | $63,530 | | | | | | |
| * adjusted to 2018 dollars | |  |  |  |  |  |  |  |
| ~ used for high cost estimate in sensitivity analysis | |  |  |  |  |  |  |  |

|  |  |  |  |  |  |  |  |  |
| --- | --- | --- | --- | --- | --- | --- | --- | --- |
| **Table S12. Breast Cancer** | | | | | | | | |
| Exposure | Percentile range of exposure | 0-9 | 10-24 | 25-49 | 50-74 | 75-89 | 90-99 | >99 |
| PFOA~ | Assigned exposure concentration (ng/mL) | 0.00 | 0.47 | 0.67 | 0.97 | 1.47 | 2.37 | 5.17 |
|  | Threshold of exposure | 0.1 ng/mL | | | | | | |
|  | OR of breast cancer | 1.00 | 1.09 | 1.14 | 1.22 | 1.37 | 1.69 | 3.23 |
|  | Relative risk of breast cancer | 1.00 | 1.09 | 1.14 | 1.22 | 1.37 | 1.68 | 3.14 |
|  | Incremental increase of breast cancer | 0.00 | 0.01 | 0.03 | 0.05 | 0.05 | 0.06 | 0.02 |
|  | Attributable fraction of breast cancer | 3.65% | | | | | | |
|  | Attributable cases of breast cancer | 3095 | | | | | | |
|  | Cost of first 6 months after breast cancer diagnosis | $159,375,251 | | | | | | |
|  | Indirect cost of attributable breast cancer as DALY lost over 10 years | $3,915,512,989 | | | | | | |
| PFOS | Assigned exposure concentration (ng/mL) | 0.00 | 1.10 | 1.70 | 2.60 | 3.90 | 5.70 | 11.9 |
|  | Threshold of exposure | 1 ng/mL | | | | | | |
|  | OR of breast cancer | 1.00 | 1.00 | 1.01 | 1.03 | 1.06 | 1.10 | 1.24 |
|  | Relative risk of breast cancer | 1.00 | 1.00 | 1.01 | 1.03 | 1.06 | 1.10 | 1.24 |
|  | Incremental increase of breast cancer | 0.00 | 0.00 | 0.00 | 0.01 | 0.01 | 0.01 | 0.00 |
|  | Attributable fraction of breast cancer | 0.50% | | | | | | |
|  | Attributable cases of breast cancer | 421 | | | | | | |
|  | Cost of first 6 months after breast cancer diagnosis | $21,692,276 | | | | | | |
|  | Indirect cost of attributable breast cancer as DALY lost over 10 years | $532,933,369 | | | | | | |
|  | Cost per case for first 6 months of breast cancer diagnosis* | $51,498 | | | | | | |
| * adjusted to 2018 dollars | |  |  |  |  |  |  |  |
| ~ used for high cost estimate in sensitivity analysis | |  |  |  |  |  |  |  |

|  |  |  |  |  |  |  |  |  |
| --- | --- | --- | --- | --- | --- | --- | --- | --- |
| **Table S13. Pneumonia** | | | | | | | | |
| Exposure | Percentile range of exposure | 0-9 | 10-24 | 25-49 | 50-74 | 75-89 | 90-99 | >99 |
| PFOA | Assigned exposure concentration (ng/mL) | 0.00 | 0.47 | 0.67 | 0.97 | 1.47 | 2.37 | 5.17 |
|  | Threshold of exposure | 1 ng/mL | | | | | | |
|  | Relative risk of pneumonia | 1.00 | 1.00 | 1.00 | 1.00 | 1.12 | 1.39 | 2.71 |
|  | Incremental increase of pneumonia | 0.00 | 0.00 | 0.00 | 0.00 | 0.02 | 0.03 | 0.02 |
|  | Attributable fraction of pneumonia | 0.58% | | | | | | |
|  | Attributable cases of pneumonia in 0-3 year olds | 447 | | | | | | |
|  | Cost of hospitalization | $1,320,147 | | | | | | |
|  | Cost of parental absenteeism associated with hospitalization | $166,169 | | | | | | |
| PFOS~ | Assigned exposure concentration (ng/mL) | 0.00 | 1.10 | 1.70 | 2.60 | 3.90 | 5.70 | 11.9 |
|  | Threshold of exposure | 0.1 ng/mL | | | | | | |
|  | Relative risk of pneumonia | 1.00 | 1.20 | 1.34 | 1.58 | 2.00 | 2.78 | 8.60 |
|  | Incremental increase of pneumonia | 0.00 | 0.03 | 0.08 | 0.13 | 0.13 | 0.14 | 0.07 |
|  | Attributable fraction of pneumonia | 8.81% | | | | | | |
|  | Attributable cases of pneumonia in 0-3 year olds | 6759 | | | | | | |
|  | Cost of hospitalization | $19,950,884 | | | | | | |
|  | Cost of parental absenteeism associated with hospitalization | $2,511,245 | | | | | | |
|  | Cost of hospitalization per case* | $2,952 | | | | | | |
|  | Cost of parental absenteeism associated with hospitalization* | $372 | | | | | | |
| * adjusted to 2018 dollars | |  |  |  |  |  |  |  |
| ~ used for high cost estimate in sensitivity analysis | |  |  |  |  |  |  |  |

| **Table S14. Example of linearization of OR for adult obesity** | |  |  |  |  |  |  |
| --- | --- | --- | --- | --- | --- | --- | --- |
| PFOS |  |  |  |  |  |  |  |
| **Percentile of Exposure** | **0-9** | **10-24** | **25-49** | **50-74** | **75-89** | **90-99** | **99-100** |
| Exposure lower limit | 0.00 | 1.60 | 2.70 | 4.70 | 7.80 | 12.0 | 26.2 |
| Exposure upper limit | 1.60 | 2.70 | 4.70 | 7.80 | 12.0 | 26.2 | - |
| Midpoint of exposures | 0.80 | 2.15 | 3.70 | 6.25 | 9.90 | 19.1 | 41.7 |
| Kg gained based on tertile of exposure (Liu, et al. 2018) | 1.50 | 1.50 | 1.50 | 1.50 | 1.50 | 1.50 | 3.50 |
| Linearized relationship | Kg gained = 0.0747*(PFOS percentile exposure midpoint) + 1.1987 | | | | | | |
| Linearized kg gained | 1.26 | 1.36 | 1.48 | 1.67 | 1.94 | 2.63 | 4.31 |

| **Table S15: Summary of findings from risk-of-bias assessments for review articles** | | | | | | | |
| --- | --- | --- | --- | --- | --- | --- | --- |
| **First author** | **Disease outcome** | **Domain 1: Study eligibility criteria** | **Domain 2: Identification and selection of studies** | **Domain 3: Data collection and study appraisal** | **Domain 4: Synthesis and findings** | **Concerns** | **Overall risk of bias** |
| Steenland | Low birth weight | Low concerns | Unclear | Unclear | Low concerns | Methods minimally described; only searched PubMed; no risk-of-bias analysis, did not identify who reviewed articles or extracted data | Low risk of bias |
| Liu | Child obesity | Low concerns | Low concerns | Low concerns | Low concerns |  | Low risk of bias |
| Bartell | Kidney and testicular cancer | Low concerns | Unclear | Unclear | Low concerns | Domains 1-3 based on Steenland and Winquist (2021), from which Bartell et al. got their articles (only searched PubMed; no risk-of-bias analysis, did not identify who reviewed articles or extracted data); Domain 4 based on Bartell; only 4 studies for kidney cancer and 2 studies for testicular cancer | Low risk of bias |
| Kim | Hypothyroidism | Low concerns | Low concerns | Low concerns | Low concerns |  | Low risk of bias |

| **Table S16: Summary of findings from risk-of-bias assessments for individual studies** | | | | | | | | | | |  |
| --- | --- | --- | --- | --- | --- | --- | --- | --- | --- | --- | --- |
| **First author** | **Disease outcome** | **Study type** | **Did selection of study participants result in appropriate comparison groups?** | **Did the study design or analysis account for important confounding and modifying variables?** | **Were outcome data complete without attrition or exclusion from analysis?** | **Can we be confident in the exposure characterization?** | **Can we be confident in the outcome assessment?** | **Were all measured outcomes reported?** | **Were there no other potential threats to internal validity (e.g., statistical methods were appropriate and researchers adhered to the study protocol)?** | **Concerns** | |
| Meng | Low birth weight | Cohort | Definitely low risk of bias | Definitely low risk of bias | Definitely low risk of bias | Definitely low risk of bias | Definitely low risk of bias | Definitely low risk of bias | Probably low risk of bias | Potential live birth bias | |
| Lauritzen | Child obesity | Cohort | Definitely low risk of bias | Definitely low risk of bias | Definitely low risk of bias | Definitely low risk of bias | Definitely low risk of bias | Definitely low risk of bias | Definitely low risk of bias |  | |
| Wen | Hypothyroidism | Cross-sectional | Definitely low risk of bias | Definitely low risk of bias | Definitely low risk of bias | Definitely low risk of bias | Definitely low risk of bias | Definitely low risk of bias | Definitely low risk of bias |  | |
| Liu | Adult obesity | Cohort | Definitely low risk of bias | Definitely low risk of bias | Definitely low risk of bias | Definitely low risk of bias | Definitely low risk of bias | Definitely low risk of bias | Probably low risk of bias | Analysis of data collected as part of RCT, so not generalizable | |
| Sun | Type 2 diabetes | Nested case-control | Definitely low risk of bias | Definitely low risk of bias | Definitely low risk of bias | Definitely low risk of bias | Definitely low risk of bias | Definitely low risk of bias | Definitely low risk of bias |  | |
| Zhang | Gestational diabetes | Cohort | Definitely low risk of bias | Definitely low risk of bias | Definitely low risk of bias | Definitely low risk of bias | Definitely low risk of bias | Definitely low risk of bias | Probably low risk of bias | Potential live birth bias | |
| Buck Louis | Endometriosis | Cross-sectional | Definitely low risk of bias | Definitely low risk of bias | Definitely low risk of bias | Definitely low risk of bias | Definitely low risk of bias | Definitely low risk of bias | Definitely low risk of bias |  | |
| Vagi | Polycystic ovarian syndrome | Case-control | Definitely low risk of bias | Definitely low risk of bias | Definitely low risk of bias | Definitely low risk of bias | Definitely low risk of bias | Definitely low risk of bias | Definitely low risk of bias |  | |
| Whitworth | Infertility | Cohort | Definitely low risk of bias | Definitely low risk of bias | Definitely low risk of bias | Definitely low risk of bias | Definitely low risk of bias | Definitely low risk of bias | Probably low risk of bias | Potential conception bias | |
| Wielsoe | Breast cancer | Case-control | Definitely low risk of bias | Definitely low risk of bias | Definitely low risk of bias | Definitely low risk of bias | Definitely low risk of bias | Definitely low risk of bias | Definitely low risk of bias |  | |
| Impinen | Child pneumonia | Cohort | Definitely low risk of bias | Definitely low risk of bias | Definitely low risk of bias | Definitely low risk of bias | Definitely low risk of bias | Definitely low risk of bias | Definitely low risk of bias | Used IPW to adjust for potential LTF bias, but did not account for live birth bias | |

| **Figure S1** |  |  |  |  |  |  |  |
| --- | --- | --- | --- | --- | --- | --- | --- |
